# Kinesin‐7 CENP‐E mediates centrosome organization and spindle assembly to regulate chromosome alignment and genome stability

**DOI:** 10.1111/cpr.13745

**Published:** 2024-09-12

**Authors:** Jie Chen, Shan Wu, Jie‐Jie He, Yu‐Peng Liu, Zhao‐Yang Deng, Han‐Kai Fang, Jian‐Fan Chen, Ya‐Lan Wei, Zhen‐Yu She

**Affiliations:** ^1^ Department of Cell Biology and Genetics, The School of Basic Medical Sciences Fujian Medical University Fuzhou Fujian China; ^2^ Key Laboratory of Stem Cell Engineering and Regenerative Medicine Fujian Province University Fuzhou Fujian China; ^3^ Medical Research Center Fujian Maternity and Child Health Hospital Fuzhou Fujian China; ^4^ College of Clinical Medicine for Obstetrics & Gynecology and Pediatrics Fujian Medical University Fuzhou Fujian China

## Abstract

Chromosome congression and alignment are essential for cell cycle progression and genomic stability. Kinesin‐7 CENP‐E, a plus‐end‐directed kinesin motor, is required for chromosome biorientation, congression and alignment in cell division. However, it remains unclear how chromosomes are aligned and segregated in the absence of CENP‐E in mitosis. In this study, we utilize the CRISPR‐Cas9 gene editing method and high‐throughput screening to establish *CENP‐E* knockout cell lines and reveal that *CENP‐E* deletion results in defects in chromosome congression, alignment and segregation, which further promotes aneuploidy and genomic instability in mitosis. Both CENP‐E inhibition and deletion lead to the dispersion of spindle poles, the formation of the multipolar spindle and spindle disorganization, which indicates that CENP‐E is necessary for the organization and maintenance of spindle poles. In addition, *CENP‐E* heterozygous deletion in spleen tissues also leads to the accumulation of dividing lymphocytes and cell cycle arrest in vivo. Furthermore, *CENP‐E* deletion also disrupts the localization of key kinetochore proteins and triggers the activation of the spindle assembly checkpoint. In summary, our findings demonstrate that CENP‐E promotes kinetochore‐microtubule attachment and spindle pole organization to regulate chromosome alignment and spindle assembly checkpoint during cell division.

## Graphical abstract

*CENP‐E* deletion results in chromosome misalignment, spindle pole disorganization and chromosome instability in mitosis. CENP‐E is essential for kinetochore‐microtubule attachment, spindle pole organization and the regulation of spindle assembly checkpoint during cell division.

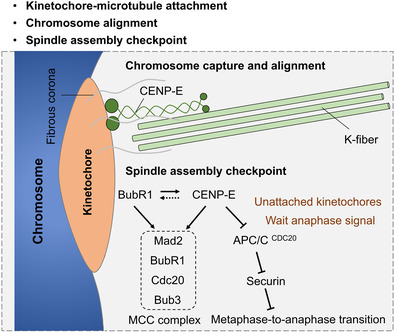

## 1 INTRODUCTION

Accurate chromosome alignment and segregation are critical for chromosome stability and faithful genome inheritance in metazoans.^1^, ^2^ Mitotic kinesins are key regulators in microtubule dynamics, spindle organization, chromosome biorientation, alignment and segregation in cell division.^3^, ^4^ Kinesin‐7 Centromere‐associated protein E (CENP‐E) is a large centromere‐associate kinesin motor required for chromosome alignment, which accumulates at the late G_2_ phase, functions in mitosis, and is specifically degraded at the end of mitosis.^5^, ^6^, ^7^, ^8^, ^9^, ^10^ During mitosis, CENP‐E plays a key role in kinetochore‐microtubule attachment, chromosome alignment and the regulation of spindle assembly checkpoint.^5^, ^11^, ^12^, ^13^, ^14^, ^15^, ^16^, ^17^ Depletion of *CENP‐E* using antibody microinjection,^5^, ^18^, ^19^ antisense oligonucleotides,^11^ siRNA^20^, ^21^, ^22^, ^23^ or chemical inhibition^24^, ^25^ results in chromosome misalignment and the activation of mitotic checkpoint in cell cycle. Although the functions of CENP‐E have been extensively studied, the phenotypes and mechanisms resulting from complete knockout of *CENP‐E* remain unknown, because *CENP‐E* knockout usually leads to cell cycle arrest and cell death.

In mice, homozygous deletion of *CENP‐E* in mice leads to early developmental arrest and embryonic lethality due to mitotic chromosome misalignment,^26^, ^27^ which is an obstacle to the study of CENP‐E in cell biology. Heterozygous deletion of *CENP‐E* also results in the missegregation of several chromosomes in the cell cycle and the development of spontaneous spleen and lung tumours.^28^, ^29^ CENP‐E co‐localizes with kinetochores during prometaphase, serves as a linker between chromosomes and the kinetochore fibres, and plays a key role in chromosome transport and end‐on attachment.^5^, ^11^, ^29^, ^30^, ^31^ Accumulating studies indicate that chromosome alignment and segregation are coordinated by the synergistic actions of multiple proteins, including CENP‐E, Dynein, KIF2C and Aurora B.^16^, ^32^, ^33^, ^34^

The kinetochore serves as a structural platform for kinetochore‐microtubule attachment and functions as a signalling hub for spindle assembly checkpoint and the regulation of metaphase‐to‐anaphase transition.^35^, ^36^, ^37^ The spindle assembly checkpoint is activated by erroneously attached kinetochores on misaligned chromosomes, which generates a ‘wait anaphase’ signalling cascade that suppresses Cdc20, a key cofactor for the anaphase‐promoting complex/cyclosome (APC/C).^38^, ^39^ BubR1 associates with Bub3, Mad2 and Cdc20 to assemble the mitotic checkpoint complex (MCC), which suppresses APC/C.^10^, ^40^, ^41^ BubR1 is required for the initial and rapid recruitment of CENP‐E to kinetochores in chromosome alignment and spindle assembly checkpoint activation.^42^, ^43^ CENP‐E promotes mitotic checkpoint signalling through the activation of BubR1 kinase activity, which is silenced by kinetochore‐microtubule capture.^13^, ^27^, ^44^, ^45^ However, the activation or silence of spindle assembly checkpoint after CENP‐E genetic deletion remains largely unknown.

Here, we combined CRISPR‐Cas9 gene editing, high‐throughput screening, chemical inhibition, high‐resolution confocal microscopy and transmission electron microscopy to address the functions of CENP‐E as a key regulator of chromosome alignment and stability. We show that both CENP‐E inhibition and deletion result in chromosome misalignment, spindle disorganization and defects in chromosome segregation. *CENP‐E* deletion leads to defects in chromosome congression and alignment, which further triggers the activation of spindle assembly checkpoint and cell cycle arrest. Taken together, our data reveal detailed functions and molecular basis of CENP‐E in chromosome alignment and cell cycle progression during cell division.

## 2 MATERIALS AND METHODS

### 2.1 Animals and ethics statements

Animal experiments were approved by the Institutional Animal Care and Use Committee at Fujian Medical University, China (Protocol No. IACUC FJMU2023‐Y‐0374). All experiments were carried out according to the Guide for Care and Use of Laboratory Animals of the National Institute of Health (NIH Publications No. 8023, revised 1978). The *CENP‐E* ^*Flox/+*^ mice (Cat# T008554) were purchased from GemPharmatech (Jiangsu, China). The *Stra8*‐*iCre* mice (Cat# T009409) were purchased from GemPharmatech (Jiangsu, China). *CENP‐E* ^*Flox/+*^ mice were crossed with *Stra8‐iCre* ^+/+^ mice to obtain *CENP‐E* ^*Flox/+*^; *Stra8‐iCre* ^+/−^ mice. The 8‐week‐old male *CENP‐E* ^*Flox/+*^; *Stra8‐iCre* ^+/−^ mice were then crossed with 8‐week‐old female wild‐type C57BL/6JGpt mice to obtain *CENP‐E* ^*null/+*^ mice (referred to as *CENP‐E* ^*+/−*^ heterozygous mice).

### 2.2 Cell culture, maintenance and treatment

HeLa cells (Cat# CCL‐2; American Type Culture Collection, ATCC) were cultured and maintained in complete DMEM/high‐glucose (C11995500BT; Gibco) supplemented with 10% fetal bovine serum (16140089; Gibco) and 1% penicillin–streptomycin solution (SV30010; Hyclone) at 37°C with 5% CO_2_ in a humidified incubator (HF90/HF240; Heal Force). For CENP‐E inhibition, GSK923295 (HY‐10299; MedChemExpress) was added in DMEM medium at a final concentration of 50 or 400 nM as indicated for 48 h and harvested for further analysis. In the control group, 0.004% DMSO were added in DMEM medium. For cell transfection, cells were transfected with the Lipofectamine 3000 reagent (L3000008; Thermo Fisher Scientific).

### 2.3 CRISPR‐Cas9 gene editing system

Mutant generation and endogenous targeting of the *CENP‐E* gene were performed by the CRISPR‐Cas9‐based gene editing method. sgRNAs were designed using an online CRISPR design tool (http://crispor.tefor.net/). The single‐strand DNA oligos were synthesized, phosphorylated and annealed using the T4 polynucleotide kinase (TaKaRa). The annealed oligonucleotides were ligated and cloned into a pX458 plasmid (pSpCas9(BB)‐2A‐GFP, Addgene No. 48138) using the *Bbs*I restriction endonuclease (R3539; NEB). The sgRNA oligos are listed in Table S1.

HeLa cells were transfected with 1 μg pX458/sgRNA plasmid and 1.5 μL Lipofectamine 3000 reagents (L3000008; Thermo Fisher Scientific). HeLa cells were then cultured for 48 h and digested using 0.25% Trypsin–EDTA (Gibco). Cells were diluted into the 96‐well plates at a density of one cell per well. Single‐cell clones were selected and cultured for 21 days. Genomic DNA extraction was performed using the column animal genomic DNA purification kit (B518251; Sangon Biotech). Growing colonies were examined using PCR amplifications for *CENP‐E* gene frameshift. DNA was ligated into the pMD18T vector (6011; TaKaRa) and cloned into the competent DH5α *Escherichia coli* cells (B528413; Sangon Biotech) for Sanger sequencing. The primers used for genotyping of HeLa cells are listed in Table S2.

### 2.4 Cell viability and crystal violet staining

For the MTT assay, cells were grown to an 80%–90% density in a 24‐well plate and harvested using 0.25% Trypsin–EDTA. Cells were incubated with PBS at 37°C for 2 min. Cells were incubated with 10 μL MTT solution using the CellTiter 96 aqueous one solution cell proliferation assay (Promega, Cat. G3580). Absorbance value (*A*_490nm_) was recorded using a microplate reader (Cat# 180316F; BioTek).

For crystal violet staining, cells were fixed in 1% paraformaldehyde in PBS (pH 7.4) at room temperature for 10 min. Cells were stained with 0.1% crystal violet staining solution for 15 min, washed three times with PBS and photographed for recording. Cells were incubated with 10% acetic acid for 2 min. Absorbance value (*A*_600nm_) was recorded using a microplate reader (Cat# 180316F; BioTek).

### 2.5 Histology, immunofluorescence and confocal imaging

Cells were seeded into a 12 mm glass coverslip (CITOGLAS) in each well of a 24‐well plate (Corning). Cells were fixed with 4% paraformaldehyde in PBS (137 mM NaCl, 2.7 mM KCl, 10 mM Na_2_HPO_4_ and 1.8 mM KH_2_PO_4_; pH 7.4) for 10 min at room temperature, washed three times with PBS and then permeabilized with 0.25% Triton X‐100 in PBS for 10 min at room temperature. Cells were washed three times with PBS and then incubated with 1% BSA in PBST for 1 h. Cells were incubated with primary antibodies in PBS with 1% BSA for 12 h at 4°C. Cells were washed three times with PBS and incubated with secondary Alexa Fluor 488/555 conjugated antibodies (Thermo Fisher Scientific) for 2 h at room temperature. Cells were washed three times with PBS and then incubated with DAPI (C1006; Beyotime) for 5 min. Cells were mounted with an antifade mounting medium (P0131; Beyotime). In negative controls, the primary antibody was omitted in the control for the validation of non‐specific fluorescence.

For haematoxylin–eosin (HE) staining, immunofluorescence of tissues, transmission electron microscopy, and TUNEL assay, see Supporting Information for more details. Images were acquired with a Nikon ECLIPSE Ti2 Series‐AXE R (Apo TIRF 60×/NA 1.49) and NIS‐elements viewer software (Nikon).

### 2.6 Flow cytometry

For cell cycle analysis, cells were harvested with 0.25% Trypsin–EDTA at 37°C for 2 min and centrifuged at 1000 × g for 5 min. Cells were fixed in cold 70% ethanol at 4°C for 16 h. Cells were centrifuged at 1000 × g for 5 min and then stained with the propidium iodide (PI) solution (50 μg/mL PI, 100 μg/mL RNase A and 0.2% Triton X‐100 in PBS) at 37°C for 30 min. Data were acquired on a flow cytometer (BD FACSCantoTMII) according to the manufacturer's protocols. For each flow cytometry plot, 10,000 cells were counted and analysed. Data analysis of DNA content and cell cycle was performed using the Modfit LT32 software (Verity Software House).

For cell apoptosis analysis, cells were harvested with 0.25% Trypsin–EDTA at 37°C for 2 min and centrifuged at 1000 × g for 5 min. Cells were resuspended in PBS and incubated with the Annexin‐FITC solution using the Annexin‐FITC apoptosis detection kit (C1062L; Beyotime). Cells were then incubated with the PI solution for 30 min. Data were acquired and analysed on a flow cytometer (BD FACSCantoTMII).

### 2.7 Statistical analysis

For each experiment, we performed at least three independent biological repetitions. Statistical analyses were performed using the GraphPad Prism 8.0 (GraphPad Software). The unpaired Student's *t*‐test or ANOVA Dunnett's multiple comparisons test was used. Results are presented as mean ± SEM. Ns, not significant; *, *p* < 0.05; **, *p* < 0.01; ***, *p* < 0.001; ****, *p* < 0.0001.

## 3 RESULTS

### 3.1 CENP‐E inhibition results in chromosome misalignment, CENP‐E mislocalization and the formation of multipolar spindles

**FIGURE 1:**
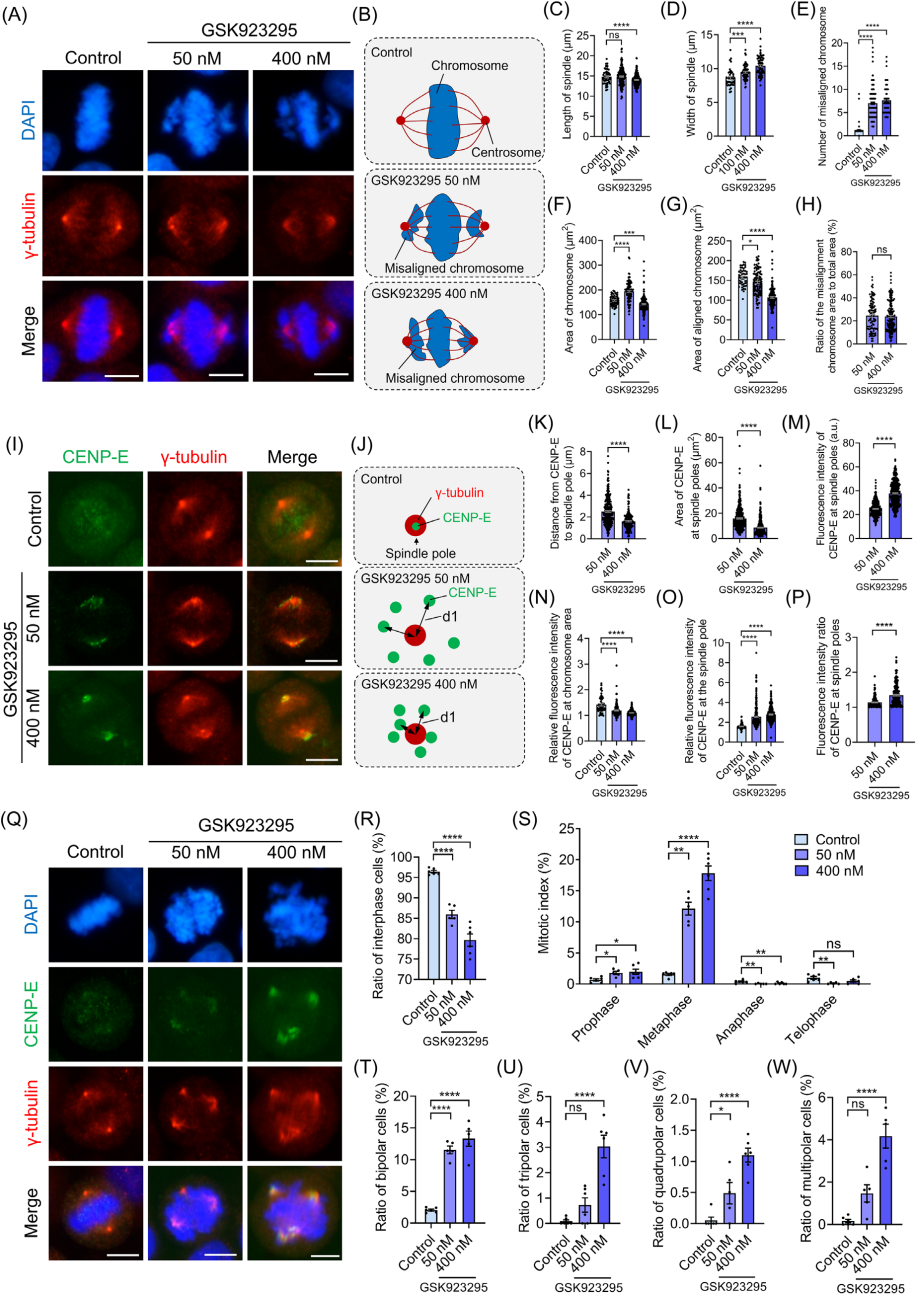
CENP‐E inhibition resulted in chromosome misalignment and the abnormal localization of CENP‐E proteins at metaphase. (A) Immunofluorescence images of γ‐tubulin in the control and GSK923295 groups. HeLa cells were treated with 50 and 400 nM GSK923295 for 24 h and then harvested for analysis. γ‐tubulin, red; DAPI, blue. Scale bar, 10 μm. (B) Schematic diagram of chromosome alignment after the incubation with different concentrations of GSK923295 in HeLa cells. (C) The length of the mitotic spindle (μm). Control, *N* = 54. GSK923295 50 nM, *N* = 206. GSK923295 400 nM, *N* = 220. (D) The width of the mitotic spindle (μm). Control, *N* = 41. GSK923295 100 nM, *N* = 81. GSK923295 400 nM, *N* = 72. (E) Numbers of misaligned chromosomes. Control, *N* = 79. GSK923295 50 nM, *N* = 143. GSK923295 400 nM, *N* = 69. (F) The area of chromosomes (μm^2^). Control, *N* = 51. GSK923295 50 nM, *N* = 101. GSK923295 400 nM, *N* = 162. (G) The area of aligned chromosomes (μm^2^). Control, *N* = 51. GSK923295 50 nM, *N* = 101. GSK923295 400 nM, *N* = 162. (H) The ratio of the misalignment chromosome area to total area (%). GSK923295 50 nM, *N* = 101. GSK923295 400 nM, *N* = 163. (I) Immunofluorescence images of CENP‐E and γ‐tubulin in the control and GSK923295 groups. γ‐tubulin, red; CENP‐E, green; DAPI, blue. Scale bar, 10 μm. (J) Patterns of relative positions of CENP‐E and centrosomes. d1 indicates the distance from CENP‐E proteins to the centrosomes. (K) Quantifications of the distances from CENP‐E proteins to spindle poles. GSK923295 50 nM, *N* = 301. GSK923295 400 nM, *N* = 151. (L) The area of CENP‐E proteins at the spindle poles (μm^2^). GSK923295 50 nM, *N* = 390. GSK923295 400 nM, *N* = 404. (M) Relative fluorescence intensity of CENP‐E at chromosome area. Control, *N* = 51. GSK923295 50 nM, *N* = 135. GSK923295 400 nM, *N* = 219. (N) Fluorescence intensity of CENP‐E proteins at the spindle poles (a.u.). GSK923295 50 nM, *N* = 390. GSK923295 400 nM, *N* = 404. (O) Relative fluorescence intensity of CENP‐E at the spindle poles. Control, *N* = 88. GSK923295 50 nM, *N* = 301. GSK923295 400 nM, *N* = 352. (P) Fluorescence intensity ratio of CENP‐E at the spindle poles (FL_spindle pole 1_/FL_spindle pole 2_). GSK923295 50 nM, *N* = 195. GSK923295 400 nM, *N* = 202. (Q) Immunofluorescence images of γ‐tubulin and CENP‐E in the control and GSK923295 groups. γ‐tubulin, red; CENP‐E, green; DAPI, blue. Scale bar, 10 μm. (R) The ratios of interphase cells (%). Control, *N* = 3407, group = 6. GSK923295 50 nM, *N* = 2049, group = 5. GSK923295 400 nM, *N* = 2509, group = 6. (S) Mitotic index in the control and GSK923295 groups. (T) The ratios of bipolar cells (%). Control, *N* = 3407, group = 6. GSK923295 50 nM, *N* = 2049, group = 5. GSK923295 400 nM, *N* = 2509, group = 6. (U) The ratios of tripolar cells (%). Control, *N* = 3407, group = 6. GSK923295 50 nM, *N* = 2049, group = 5. GSK923295 400 nM, *N* = 2509, group = 6. (V) The ratios of quadrupolar cells (%). Control, *N* = 3407, group = 6. GSK923295 50 nM, *N* = 2049, group = 5. GSK923295 400 nM, *N* = 2509, group = 6. (W) The ratios of all multipolar cells (%). Control, *N* = 3407, group = 6. GSK923295 50 nM, *N* = 2049, group = 5. GSK923295 400 nM, *N* = 2509, group = 6. Results are expressed as mean ± SEM of more than three independent experiments. *N* values indicate the number of cells per experiment. For two‐group graphs, unpaired Student's *t*‐test. For multiple‐group graphs, ANOVA Dunnett's multiple comparisons test. ns, *p* > 0.05; *, *p* < 0.05; **, *p* < 0.01; ***, *p* < 0.001; ****, *p* < 0.0001.

CENP‐E proteins were highly expressed in the G_2_/M phase, accumulated at kinetochores during prometaphase and metaphase and translocated to the central spindle and midbody during anaphase and cytokinesis (Figure S1A–J). To investigate the functions of CENP‐E in HeLa cells, we employed the specific inhibitor GSK923295 to suppress CENP‐E in HeLa cells for 24 h, which blocks the ATPase activity of CENP‐E's motor domain and locks CENP‐E on microtubules.^24^, ^46^ CENP‐E inhibition resulted in the misalignment of several chromosomes at the spindle poles (Figure 1A,B). The length of the mitotic spindle decreased to 14.00 ± 0.10 μm in the 400 nM GSK923295 group compared with 14.81 ± 0.24 μm in the control group (Figure 1C). In addition, the width of the mitotic spindle increased following CENP‐E inhibition (Figure 1D). Statistical analysis revealed a significant increase in the number of misaligned chromosomes after CENP‐E inhibition (Figure 1E). Subsequently, we measured the area of the chromosomes and observed that CENP‐E inhibition resulted in the disorganization of chromosomes in the equatorial plate. The chromosomes were more disorganized in the 50 nM GSK923295 group compared with the 400 nM GSK923295 group (Figure 1F–H). The results indicate different degrees of CENP‐E inhibition resulted in chromosome misalignment, and the degree of alignment scattering increased with the concentration of GSK923295.

To further investigate the effects of CENP‐E inhibition, HeLa cells were labelled with the CENP‐E antibody. We found that CENP‐E proteins were accumulated at the kinetochores and localized at spindle microtubules in metaphase HeLa cells (Figure S1). Remarkably, GSK923295‐mediated CENP‐E inhibition resulted in the accumulation of CENP‐E on kinetochores of misaligned chromosomes around the spindle poles (Figure 1I,J). By comparing the fluorescence intensity of CENP‐E proteins around the spindle poles, we observed that the distance from CENP‐E to spindle poles decreased in the 400 nM group compared with the 50 nM group (Figure 1K). The area of CENP‐E at spindle poles decreased in the 400 nM group compared to the 50 nM group. These results suggest that, with increasing levels of CENP‐E inhibition, the misaligned chromosomes became more concentrated at the spindle poles (Figure 1L). Concurrently, the localization of CENP‐E at kinetochores of aligned chromosomes at the metaphase plate also diminished after CENP‐E inhibition (Figure 1I,M). We measured the fluorescence intensity of CENP‐E at spindle poles and found that CENP‐E proteins were more abundant at the misaligned kinetochores in the 400 nM group compared with the 50 nM group (Figure 1N–P).

We investigated the mitotic index of HeLa cells and observed that CENP‐E inhibition resulted in cell cycle disruption (Figure 1Q–S). The ratios of interphase cells significantly decreased in the 50 nM and 400 nM GSK923295 groups compared with the control (Figure 1R). The ratios of metaphase cells increased after CENP‐E inhibition (Figure 1S). In addition, the ratios of anaphase cells decreased after CENP‐E inhibition (Figure 1S). Notably, the ratios of bipolar cells increased significantly following CENP‐E inhibition (Figure 1T). Interestingly, we observed an increase in the ratios of multipolar cells after CENP‐E inhibition (Figure 1U–W). These results suggest that CENP‐E inhibition leads to the formation of metaphase cells and multipolar spindles in HeLa cells.

To further investigate the roles of CENP‐E in spindle assembly, we measured the area and fluorescence intensity of γ‐tubulin in the control and GSK923295 groups. CENP‐E inhibition resulted in centrosome dispersal, and the area of γ‐tubulin significantly increased (Figure S2A,B). Meanwhile, the fluorescence intensity of γ‐tubulin at the spindle poles gradually decreased after CENP‐E inhibition, indicating the dispersion of γ‐tubulin proteins in the spindle poles (Figure S2C). Furthermore, we observed an influence on the architecture of the mitotic spindle following CENP‐E inhibition (Figure S2A,D–G). Together, these findings suggest that CENP‐E plays a role in chromosome alignment and spindle organization in cell division.

### 3.2 *CENP‐E* deletion leads to chromosome misalignment and centrosome disorganization

**FIGURE 2:**
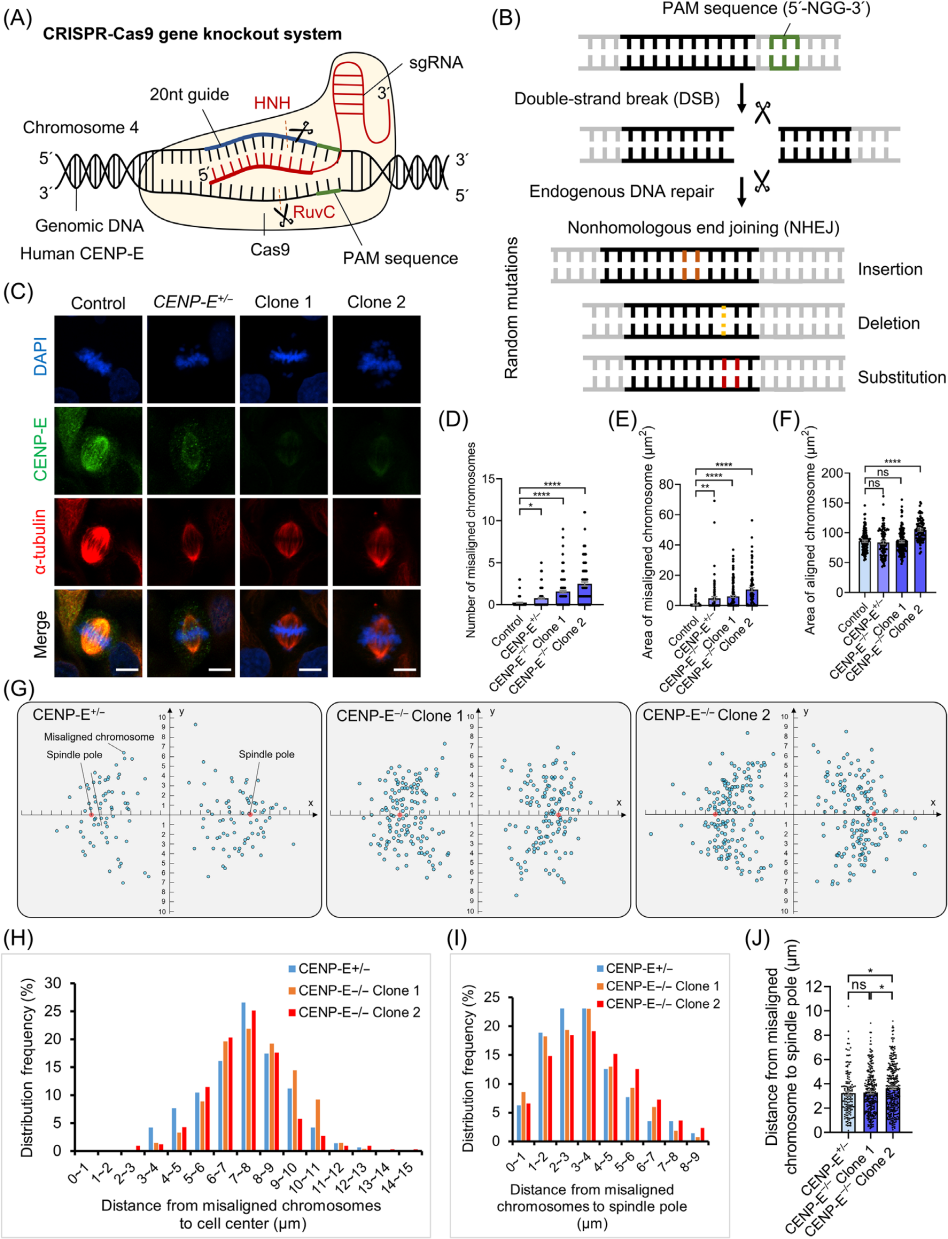
Construction of *CENP‐E* knockout HeLa cells using the CRISPR‐Cas9 gene editing system. (A) Schematic diagram of the construction of *CENP‐E* knockout HeLa cells using the CRISPR‐Cas9 gene editing system. The *Streptococcus pyogenes* Cas9 nuclease targets the human *CENP‐E* locus in genomic DNA by a sgRNA consisting of a 20‐nt guide sequence (red) and a scaffold. The gRNA paired with target DNA upstream of a requisite 5′‐NGG adjacent PAM motif. Cas9 nuclease mediates a double‐strand breakage ~3 bp upstream of the PAM motif using the HNH and RuvC domains. (B) Schematic diagram of the principle of the knockout mechanism. After cleavage by Cas9, the *CENP‐E* locus can be repaired by a nonhomologous end joining (NHEJ) pathway. This process results in random insertions, deletions or substitutions at the site of the cleavage, then usually leads to frameshifts and the formation of a premature stop codon, and finally causes gene knockout of *CENP‐E*. (C) Immunofluorescence images of α‐tubulin and CENP‐E in the control, *CENP‐E* ^*+/−*^, *CENP‐E* ^*−/−*^ Clone 1 and Clone 2 groups. α‐tubulin, red; CENP‐E, green; DAPI, blue. Scale bar, 10 μm. (D) Quantifications of the number of misaligned chromosomes. Control, *N* = 122. *CENP‐E* ^+/−^, *N* = 146. *CENP‐E* ^−/−^ Clone 1, *N* = 138. *CENP‐E* ^−/−^ Clone 2, *N* = 112. (E) The area of misaligned chromosomes (μm^2^). Control, *N* = 111. *CENP‐E* ^+/−^, *N* = 81. *CENP‐E* ^−/−^ Clone 1, *N* = 140. *CENP‐E* ^−/−^ Clone 2, *N* = 92. (F) The area of aligned chromosomes (μm^2^). Control, *N* = 111. *CENP‐E* ^+/−^, *N* = 81. *CENP‐E* ^−/−^ Clone 1, *N* = 140. *CENP‐E* ^−/−^ Clone 2, *N* = 92. (G) Distribution patterns of misaligned chromosomes in the *CENP‐E* ^*+/−*^, *CENP‐E* ^*−/−*^ Clone 1 and Clone 2 groups. (H) Distribution frequency of the distances between misaligned chromosomes and the cell centre in the *CENP‐E* ^*+/−*^, *CENP‐E* ^*−/−*^ Clone 1 and Clone 2 groups. (I) Distribution frequency of the distances from misaligned chromosomes to the cell centre in the *CENP‐E* ^*+/−*^, *CENP‐E* ^*−/−*^ Clone 1 and Clone 2 groups. (J) The distances from misaligned chromosomes to the spindle poles (μm). *CENP‐E* ^+/−^, *N* = 142. *CENP‐E* ^−/−^ Clone 1, *N* = 266. *CENP‐E* ^−/−^ Clone 2, *N* = 298. For all graphs, mean ± SEM. For all graphs, ANOVA Dunnett's multiple comparisons test. ns, *p* > 0.05; *, *p* < 0.05; **, *p* < 0.01; ***, *p* < 0.001; ****, *p* < 0.0001.

To further investigate the roles of CENP‐E in cell division, we generated *CENP‐E* completely knockout HeLa cells using the CRISPR‐Cas9 gene editing system (Figures 2A and S3A–F). HeLa cells transfected with *CENP‐E* specific sgRNA resulted in double‐strand breaks at the human genomic *CENP‐E* locus and triggered endogenous DNA repairs (Figure 2B). Nonhomologous end joining induced random mutations, which finally led to insertions, deletions or substitutions in the *CENP‐E* locus (Figure 2B). We selected three *CENP‐E* gene editing HeLa cells according to genotyping, which are referred to as the *CENP‐E* ^*+/−*^ heterozygous HeLa cells, *CENP‐E* ^*−/−*^ Clone 1 and *CENP‐E* ^*−/−*^ Clone 2 HeLa cells (Figure 2C). We found that CENP‐E proteins were completely knockout in *CENP‐E* ^*−/−*^ Clone 1 and *CENP‐E* ^*−/−*^ Clone 2 HeLa cells (Figures 2C and S3F). In addition, CENP‐E proteins were significantly decreased in *CENP‐E* ^*+/−*^ heterozygous HeLa cells (Figure 2C). Notably, *CENP‐E* deletion resulted in several chromosomes misaligned at the spindle poles (Figure 2C–F). The area of misaligned chromosomes significantly increased after *CENP‐E* deletion (Figure 2F). The distribution pattern of each misaligned chromosome in *CENP‐E* ^*+/−*^ heterozygous HeLa cells, *CENP‐E* ^*−/−*^ Clone 1 and *CENP‐E* ^*−/−*^ Clone 2 HeLa cells were normalized and calculated (Figure 2G). Statistical analysis revealed that misaligned chromosomes were distributed more dispersedly in the *CENP‐E* ^*+/‐*^HeLa cells compared with the *CENP‐E* ^*−/‐*^HeLa cells (Figure 2G,H). In the absence of CENP‐E, chromosomes were distributed closer to the centrosomes (Figure 2G,I,J). Together, these results indicate that *CENP‐E* deletion results in chromosome misalignment and several chromosomes scattered around the spindle poles.

**FIGURE 3:**
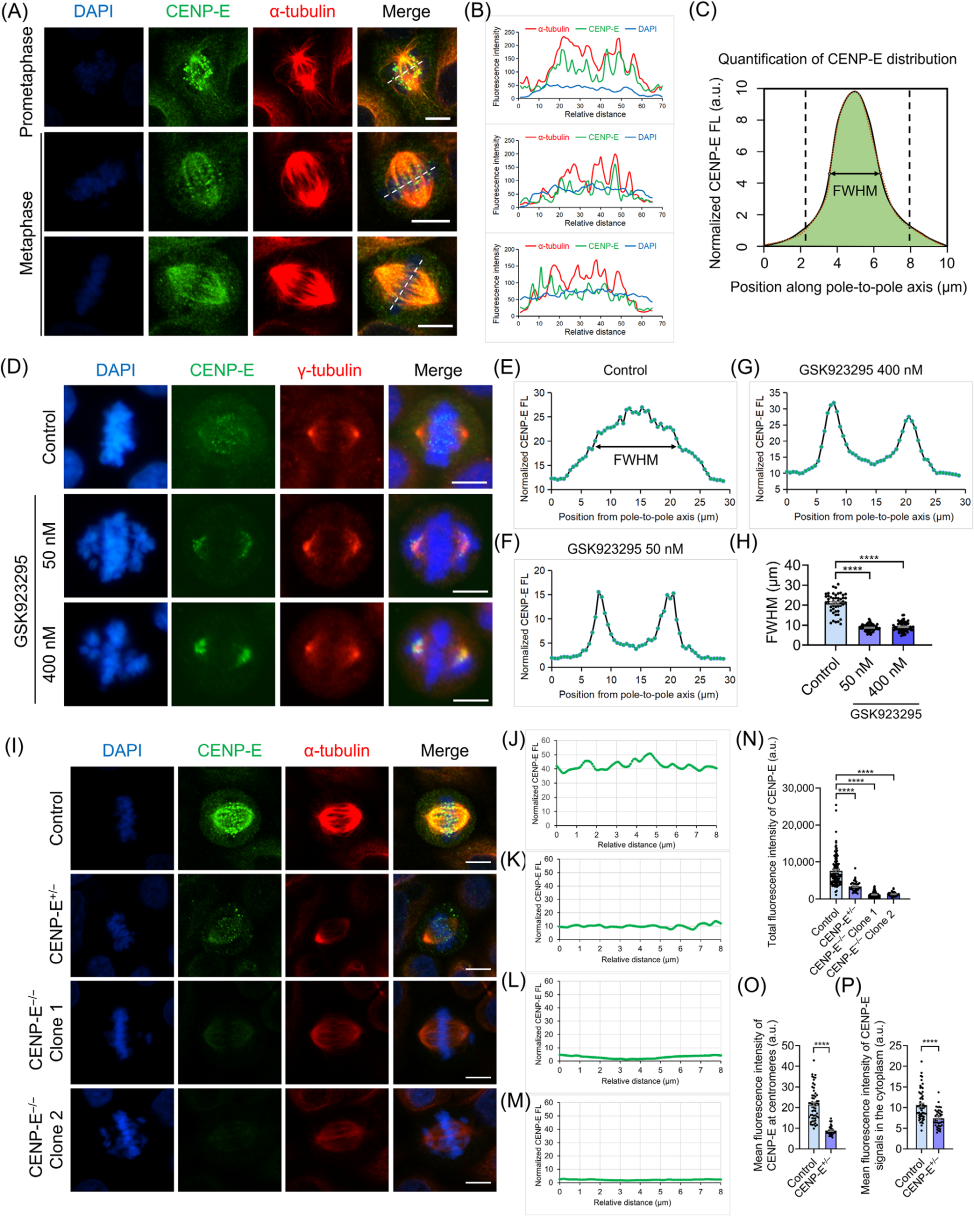
Dynamic localization patterns of CENP‐E proteins in wild‐type, GSK923295 inhibited and *CENP‐E* knockout cells. (A) Immunofluorescence images of α‐tubulin and CENP‐E in wild‐type HeLa cells. α‐tubulin, red; CENP‐E, green; DAPI, blue. Scale bar, 10 μm. CENP‐E proteins mainly accumulated at kinetochores during prometaphase and gradually disassociated from kinetochores on aligned chromosomes to k‐fibres and cytoplasm in metaphase. (B) Line‐scan analyses of fluorescence intensities of α‐tubulin and CENP‐E in HeLa cells during in prometaphase and metaphase. The X‐axis indicates the relative distance. The Y‐axis indicates the fluorescence intensity. (C) Schematic methods used to analyse fluorescence intensities of CENP‐E proteins. Distributions of CENP‐E proteins were measured using the plot profile analysis in the Image J software and the full width at half max (FWHM) of a Gaussian fit. (D) Immunofluorescence images of γ‐tubulin and CENP‐E in the control and GSK923295 groups. γ‐tubulin, red; CENP‐E, green; DAPI, blue. Scale bar, 10 μm. (E–G) Normalized CENP‐E fluorescence intensity in the control group (E), the 50 nM GSK923295 group (F) and the 400 nM GSK923295 group (G). (H) FWHM (μm). Control, *N* = 48. GSK923295 50 nM, *N* = 71. GSK923295 400 nM, *N* = 67. (I) Immunofluorescence images of α‐tubulin and CENP‐E in the control and CENP‐E knockout groups. α‐tubulin, red; CENP‐E, green; DAPI, blue. Scale bar, 10 μm. (J–M) Normalized CENP‐E fluorescence intensities in the control group (J), the *CENP‐E* ^*+/−*^ group (K), the *CENP‐E* ^*−/−*^ Clone 1 group (L), the *CENP‐E* ^*−/−*^ Clone 2 groups (M). (N) Total fluorescence intensity of CENP‐E proteins (a.u.). Control, *N* = 105. *CENP‐E* ^+/−^, *N* = 41. *CENP‐E* ^−/−^ Clone 1, *N* = 57. *CENP‐E* ^−/−^ Clone 2, *N* = 44. (O) Mean fluorescence intensity of CENP‐E proteins at centromeres (a.u.). Control, *N* = 58. *CENP‐E* ^+/−^, *N* = 41. (P) Mean fluorescence intensity of CENP‐E proteins in the cytoplasm (a.u.). Control, *N* = 56. *CENP‐E* ^+/−^, *N* = 39. For all graphs, mean ± SEM. For two‐group graphs, unpaired Student's *t*‐test. For multiple‐group graphs, ANOVA Dunnett's multiple comparisons test. ns, *p* > 0.05; *, *p* < 0.05; **, *p* < 0.01; ***, *p* < 0.001; ****, *p* < 0.0001.

During prometaphase, CENP‐E proteins mainly accumulated at the kinetochores and spindle microtubules in HeLa cells (Figure 3A,B). CENP‐E proteins gradually disassociated from the kinetochores after all chromosomes were aligned during metaphase (Figure 3A,B). We quantified the distribution of CENP‐E proteins and employed an FWHM index for the normalized CENP‐E fluorescence intensities at the equatorial plate (Figure 3C,D). Notably, we found that CENP‐E proteins largely disassociated from the kinetochores and remained at misaligned kinetochores after CENP‐E inhibition (Figure 3D–H). In the *CENP‐E* ^*+/−*^ HeLa cells, CENP‐E proteins also decreased and disassociated from the spindles and kinetochores (Figure 3I–P). Meanwhile, in *CENP‐E* ^*−/−*^ HeLa cells, CENP‐E proteins were completely knocked out (Figure 3I–P).

**FIGURE 4:**
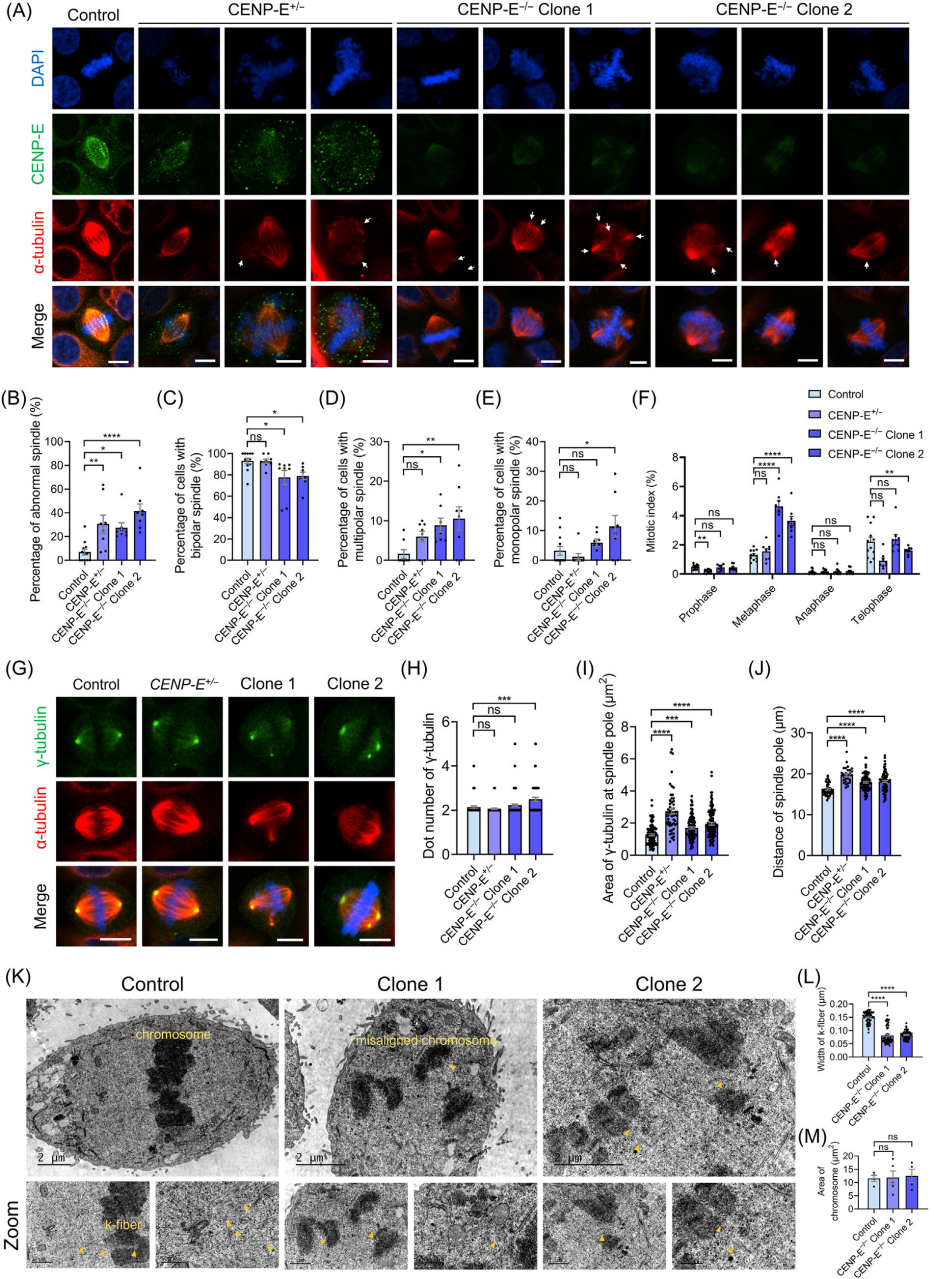
*CENP‐E* deletion resulted in the formation of multipolar spindles and the disorganization of spindle poles. (A) Immunofluorescence images of spindle structural abnormalities in the control, *CENP‐E* ^*+/−*^, *CENP‐E* ^*−/−*^ Clone 1 and Clone 2 groups. α‐tubulin, red; CENP‐E, green; DAPI, blue. Scale bar, 10 μm. (B) Percentage of abnormal spindle (%). Control, *N* = 10,913, group = 12. *CENP‐E* ^+/−^, *N* = 8250, group = 8. *CENP‐E* ^−/−^ Clone 1, *N* = 4582, group = 8. *CENP‐E* ^−/−^ Clone 2, *N* = 4119, group = 8. (C) Percentage of cells with bipolar spindle (%). Control, *N* = 10,913, group = 12. *CENP‐E* ^+/−^, *N* = 8250, group = 8. *CENP‐E* ^−/−^ Clone 1, *N* = 4582, group = 8. *CENP‐E* ^−/−^ Clone 2, *N* = 4119, group = 8. (D) Percentage of cells with multipolar spindle (%). Control, *N* = 8363, group = 8. *CENP‐E* ^+/−^, *N* = 8250, group = 8. *CENP‐E* ^−/−^ Clone 1, *N* = 4276, group = 7. *CENP‐E* ^−/−^ Clone 2, *N* = 4119, group = 8. (E) Percentage of cells with monopolar spindle (%). Control, *N* = 10,913, group = 12. *CENP‐E* ^+/−^, *N* = 8250, group = 8. *CENP‐E* ^−/−^ Clone 1, *N* = 4582, group = 8. *CENP‐E* ^−/−^ Clone 2, *N* = 4119, group = 8. (F) Mitotic index in the control and *CENP‐E* knockout groups. (G) Immunofluorescence images of α‐tubulin and γ‐tubulin in the control, *CENP‐E* ^*+/−*^, *CENP‐E* ^*−/−*^ Clone 1 and Clone 2 groups. α‐tubulin, red; γ‐tubulin, green; DAPI, blue. Scale bar, 10 μm. (H) Quantifications of the dot number of γ‐tubulin. Control, *N* = 50. *CENP‐E* ^+/−^, *N* = 59. *CENP‐E* ^−/−^ Clone 1, *N* = 133. *CENP‐E* ^−/−^ Clone 2, *N* = 176. (I) The area of γ‐tubulin at the spindle poles (μm^2^). Control, *N* = 104. *CENP‐E* ^+/−^, *N* = 53. *CENP‐E* ^−/−^ Clone 1, *N* = 120. *CENP‐E* ^−/−^ Clone 2, *N* = 128. (J) Distance of the spindle poles (μm). Control, *N* = 44. *CENP‐E* ^+/−^, *N* = 29. *CENP‐E* ^−/−^ Clone 1, *N* = 99. *CENP‐E* ^−/−^ Clone 2, *N* = 80. (K) Representative images of transmission electron micrograph in the control, *CENP‐E* ^*−/−*^ Clone 1 and Clone 2 groups. (L) Width of the k‐fibres (μm). Control, *N* = 60, group = 4. *CENP‐E* ^−/−^ Clone 1, *N* = 60, group = 5. *CENP‐E* ^−/−^ Clone 2, *N* = 60, group = 4. (M) The area of each chromosome (μm^2^). Control, *N* = 4. *CENP‐E* ^−/−^ Clone 1, *N* = 5. *CENP‐E* ^−/−^ Clone 2, *N* = 4. For all graphs, ANOVA Dunnett's multiple comparisons test. ns, *p* > 0.05; *, *p* < 0.05; **, *p* < 0.01; ***, *p* < 0.001; ****, *p* < 0.0001.

Notably, *CENP‐E* deletion led to the formation of multipolar spindles (Figure 4A–C). Statistical analysis revealed that the ratios of bipolar spindles decreased (Figure 4C), while the ratios of tripolar and multipolar spindles significantly increased after *CENP‐E* deletion (Figure 4D,E). Similarly, to the findings with GSK923295 mediated CENP‐E inhibition, *CENP‐E* deletion resulted in a significant increase in metaphase cells (Figure 4F). Additionally, the ratios of prophase, anaphase and telophase cells remained unaffected after *CENP‐E* deletion (Figure 4F). We further examined the fluorescence intensities of γ‐tubulin in *CENP‐E* knockout HeLa cells and found that the dispersion of γ‐tubulin‐labelled centrosomes following *CENP‐E* deletion (Figures 4G and S4A–P). Furthermore, *CENP‐E* siRNA knockdown also results in the disorganized spindle poles, the formation of multipolar spindle and the dispersion of γ‐tubulin‐labelled centrosomes (Figure S4Q–T). The number of γ‐tubulin dots increased after *CENP‐E* deletion (Figure 4H). Meanwhile, the area of γ‐tubulin at spindle poles significantly increased after *CENP‐E* deletion, indicating an influence of *CENP‐E* deletion on centrosome organization in HeLa cells (Figure 4I). Furthermore, the distances between two spindle poles were increased after *CENP‐E* deletion (Figure 4J). In addition, immunofluorescence of key centrosomal protein Eg5 and Aurora A also revealed that the spindle pole distance also increased after *CENP‐E* deletion (Figure S4J–P). We then performed transmission electron microscopy to observe the ultrastructure of mitotic spindles in both control and *CENP‐E* knockout HeLa cells. We observed that *CENP‐E* deletion resulted in chromosome misalignment and spindle disorganization (Figure 4K). The width of k‐fibres significantly decreased following *CENP‐E* deletion (Figure 4L). In addition, the area of each chromosome remained unaffected after *CENP‐E* deletion (Figure 4M). Together, these findings suggest that CENP‐E is essential for spindle pole organization, centrosome integrity and k‐fibre assembly during metaphase.

### 3.3 CENP‐E is essential for spindle organization, chromosome alignment and cell cycle progression in vivo

**FIGURE 5:**
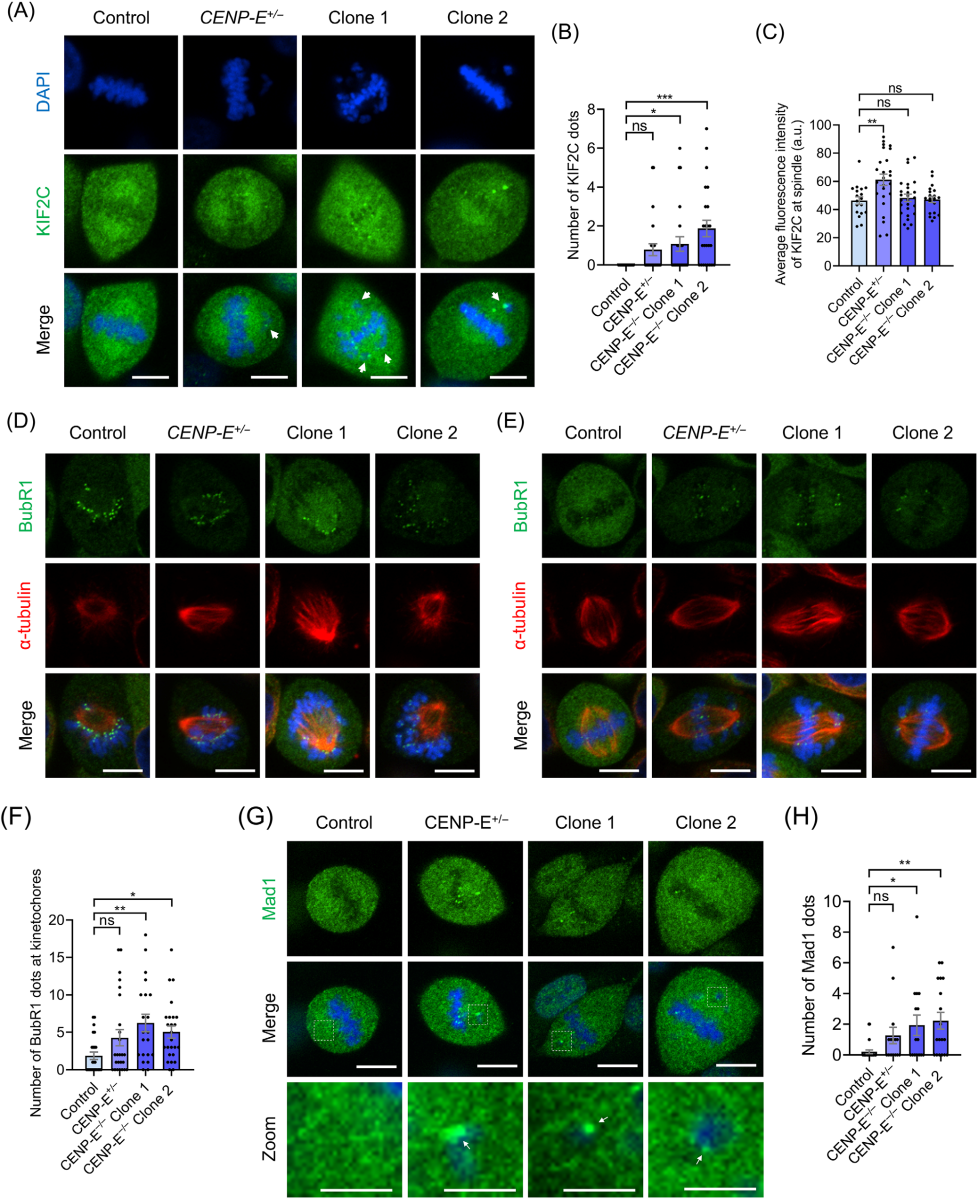
*CENP‐E* deletion led to the accumulation of KIF2C, BubR1 and Mad1 proteins at misaligned kinetochores and the activation of the spindle assembly checkpoint. (A) Immunofluorescence images of KIF2C proteins in the control, *CENP‐E* ^*+/−*^, *CENP‐E* ^*−/−*^ Clone 1 and Clone 2 groups. KIF2C, green; DAPI, blue. Scale bar, 10 μm. (B) Quantifications of the number of KIF2C dots on unaligned chromosomes. Control, *N* = 24. *CENP‐E* ^+/−^, *N* = 24. *CENP‐E* ^−/−^ Clone 1, *N* = 24. *CENP‐E* ^−/−^ Clone 2, *N* = 24. (C) Average fluorescence intensity of KIF2C proteins at the spindle (a.u.). Control, *N* = 18. *CENP‐E* ^+/−^, *N* = 25. *CENP‐E* ^−/−^ Clone 1, *N* = 26. *CENP‐E* ^−/−^ Clone 2, *N* = 20. (D) Immunofluorescence images of α‐tubulin and BubR1 in prometaphase. α‐tubulin, red; BubR1, green; DAPI, blue. Scale bar, 10 μm. (E) Immunofluorescence images of α‐tubulin and BubR1 in metaphase. α‐tubulin, red; BubR1, green; DAPI, blue. Scale bar, 10 μm. (F) Quantifications of the number of BubR1 dots at kinetochores. Control, *N* = 24. *CENP‐E* ^+/−^, *N* = 25. *CENP‐E* ^−/−^ Clone 1, *N* = 21. *CENP‐E* ^−/−^ Clone 2, *N* = 25. (G) Immunofluorescence images of Mad1 proteins in the control, *CENP‐E* ^*+/−*^, *CENP‐E* ^*−/−*^ Clone 1 and Clone 2 groups. Mad1, green; DAPI, blue. Scale bar, 10 μm. (H) Quantifications of the number of Mad1 dots on unaligned chromosomes. Control, *N* = 24. *CENP‐E* ^+/−^, *N* = 15. *CENP‐E* ^−/−^ Clone 1, *N* = 15. *CENP‐E* ^−/−^ Clone 2, *N* = 18. For all graphs, ANOVA Dunnett's multiple comparisons test. ns, *p* > 0.05; *, *p* < 0.05; **, *p* < 0.01; ***, *p* < 0.001.

Notably, we found that *CENP‐E* deletion resulted in the accumulation of KIF2C proteins on misaligned kinetochores around the spindle poles (Figure 5A). Statistical analysis revealed that the number of KIF2C signals on unattached kinetochores significantly increased after *CENP‐E* deletion (Figure 5A,B). In addition, the average fluorescence intensity of KIF2C at the spindle was not influenced in the *CENP‐E* ^*−/−*^ HeLa cells (Figure 5A,C). We observed that the localization of BubR1 proteins on kinetochores was decreased on aligned kinetochores after *CENP‐E* deletion during prometaphase (Figure 5D). However, the localization of BubR1 on misaligned kinetochores was increased after *CENP‐E* deletion in metaphase (Figure 5E,F). We stained HeLa cells with key checkpoint protein Mad1 and found that the Mad1 proteins were accumulated at misaligned kinetochores in both *CENP‐E* ^*+/−*^ and *CENP‐E* ^*+/+*^ cells (Figure 5G,H). Together, these data indicate that *CENP‐E* deletion influences the localization of key kinetochore proteins, including BubR1 and KIF2C, and activates the Mad1‐related spindle assembly checkpoint.

**FIGURE 6:**
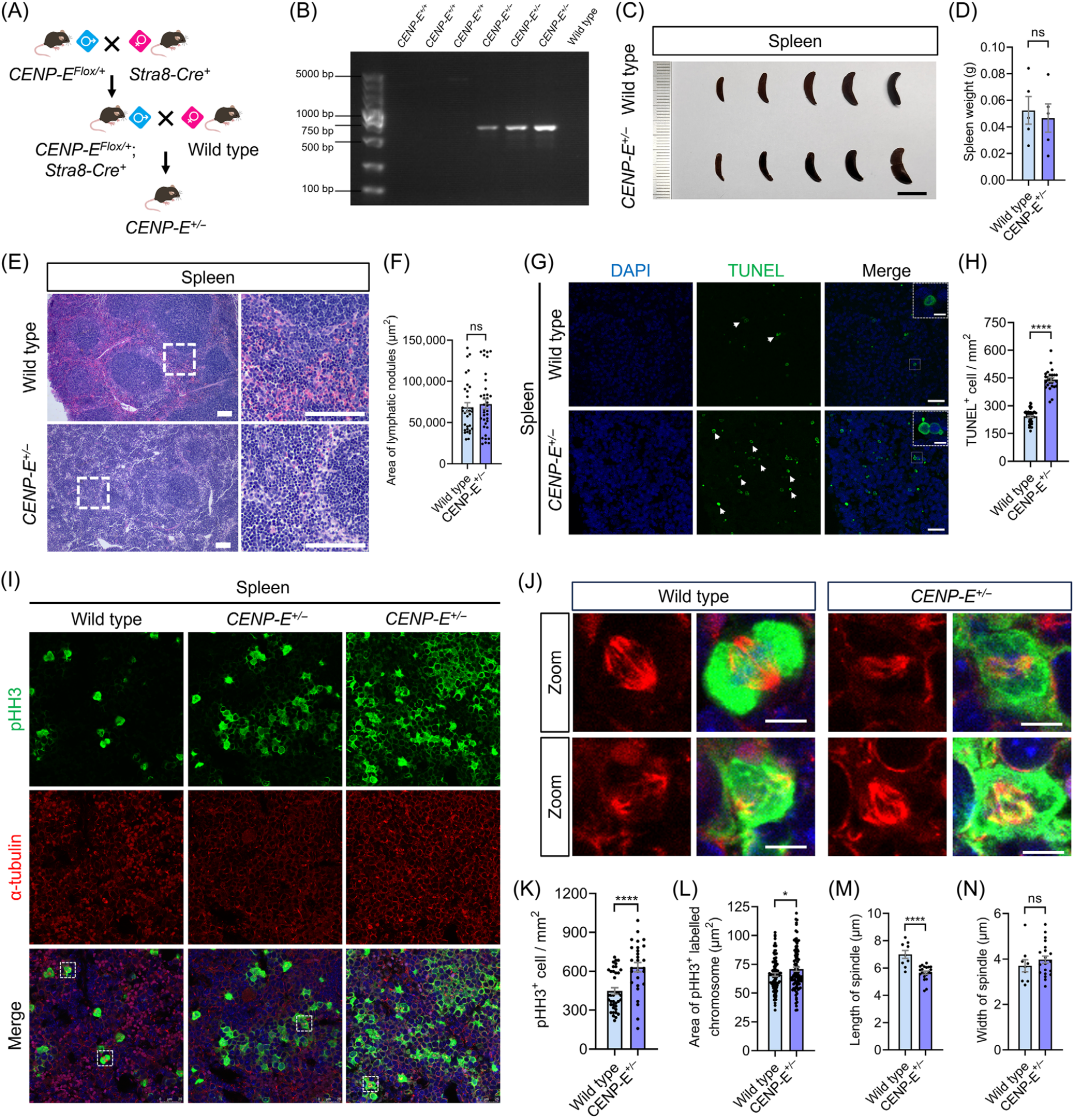
*CENP‐E* heterozygous led to the increase in dividing lymphocytes, cell cycle alteration and subsequent cell death. (A) Construction of the *CENP‐E* ^*+/−*^ mouse models. *CENP‐E* ^Flox/+^ male mice cross with *Stra8‐Cre* ^+^ female mice to harvest the *CENP‐E* ^Flox/+^; *Stra8‐Cre* ^+^ male mice. *CENP‐E* ^Flox/+^; *Stra8‐Cre* ^+^ male mice cross with wild‐type female mice to get the *CENP‐E* ^+/−^ mice. (B) Genotyping of the *CENP‐E* ^*+/−*^ mouse models. The 632 bp DNA fragment indicates the deletion of *CENP‐E* in the DNA locus. (C) Representative images of the spleen tissue in the wild type and *CENP‐E* ^*+/−*^ mice. Scale bar, 1 cm. (D) Spleen weight (g). Wild type, *N* = 5. *CENP‐E* ^+/−^, *N* = 5. (E) HE staining of the spleen tissue in the wild type and *CENP‐E* ^*+/−*^ mice. Scale bar, 100 μm. (F) Area of lymphatic nodules (μm^2^). Wild type, *N* = 31. *CENP‐E* ^+/−^, *N* = 37. (G) Immunofluorescence images of TUNEL staining of splenic cord in the spleen. TUNEL, green; DAPI, blue. Scale bar, 25 μm. (H) Relative number of TUNEL‐positive cells. Wild type, *N* = 3466, group = 45. *CENP‐E* ^+/−^, *N* = 3644, group = 26. (I) Immunofluorescence images of pHH3 and α‐tubulin in the splenic cord of the spleen tissue in the wild type and *CENP‐E* ^*+/−*^ mice. α‐tubulin, red; pHH3, green; DAPI, blue. Scale bar, 25 μm. (J) Representative immunofluorescence images of the lymphocyte division phase were shown in the zoom. α‐tubulin, red; pHH3, green; DAPI, blue. Scale bar, 5 μm. (K) Relative number of pHH3‐positive cells. Wild type, *N* = 1393, group = 38. *CENP‐E* ^+/−^, *N* = 1498, group = 29. (L) Area of pHH3‐labelled chromosomes (μm^2^). Wild type, *N* = 100. *CENP‐E* ^+/−^, *N* = 100. (M) Length of the mitotic spindle in lymphocytes during metaphase (μm). Wild type, *N* = 9. *CENP‐E* ^+/−^, *N* = 21. (N) Width of the mitotic spindle in lymphocytes during metaphase (μm). Wild type, *N* = 9. *CENP‐E* ^+/−^, *N* = 21. For all graphs, unpaired Student's *t*‐test. ns, *p* > 0.05; *, *p* < 0.05; **, *p* < 0.01; ***, *p* < 0.001; ****, *p* < 0.0001.

To further analyse the functions of CENP‐E in vivo, we generated *CENP‐E* heterozygous mice using the CRISPR‐Cas9 and Cre‐LoxP system (Figures 6A and S5A,B). We harvested *CENP‐E* ^*+/−*^ mice and validated the knockout of *CENP‐E* using genotyping (Figure 6B). Previous studies have suggested that the *CENP‐E* gene is robustly expressed in the spleen tissue.^23^ Subsequently, we harvested the spleen for further analysis. We found that the weight of the spleen remained unaffected in the *CENP‐E* ^*+/−*^ mice compared with the control (Figures 6C,D and S6A–C). Notably, *CENP‐E* deletion resulted in a significant increase in TUNEL‐positive cells in *CENP‐E* ^*+/−*^ mice compared with the control (Figures 6E–H and S6D,E), indicating that *CENP‐E* deletion induces cell apoptosis in lymphocytes. In addition, the area of lymphatic nodules was not influenced after *CENP‐E* deletion (Figure 6E,F).

We then stained spleen tissues with the pHH3 antibody and found that pHH3‐positive cells also significantly increased in *CENP‐E* ^*+/−*^ mice compared with the control (Figure 6I–K). We observed that the chromosomes were aligned at the equatorial plate and the mitotic spindle was organized in the metaphase lymphocytes in the wild‐type mice. However, the chromosomes were misaligned, and the mitotic spindle was disorganized in the *CENP‐E* ^*+/−*^ mice (Figure 6J,L–N). Statistical analysis revealed that the chromosome area was significantly increased after *CENP‐E* deletion (Figure 6J,L). Furthermore, the length of the mitotic spindle decreased after *CENP‐E* deletion, while the width of the mitotic spindle remained unaffected (Figure 6J,M,N). Together, these results suggest that *CENP‐E* deletion results in a significant increase in dividing lymphocytes and apoptotic cells in the spleen in vivo.

**FIGURE 7:**
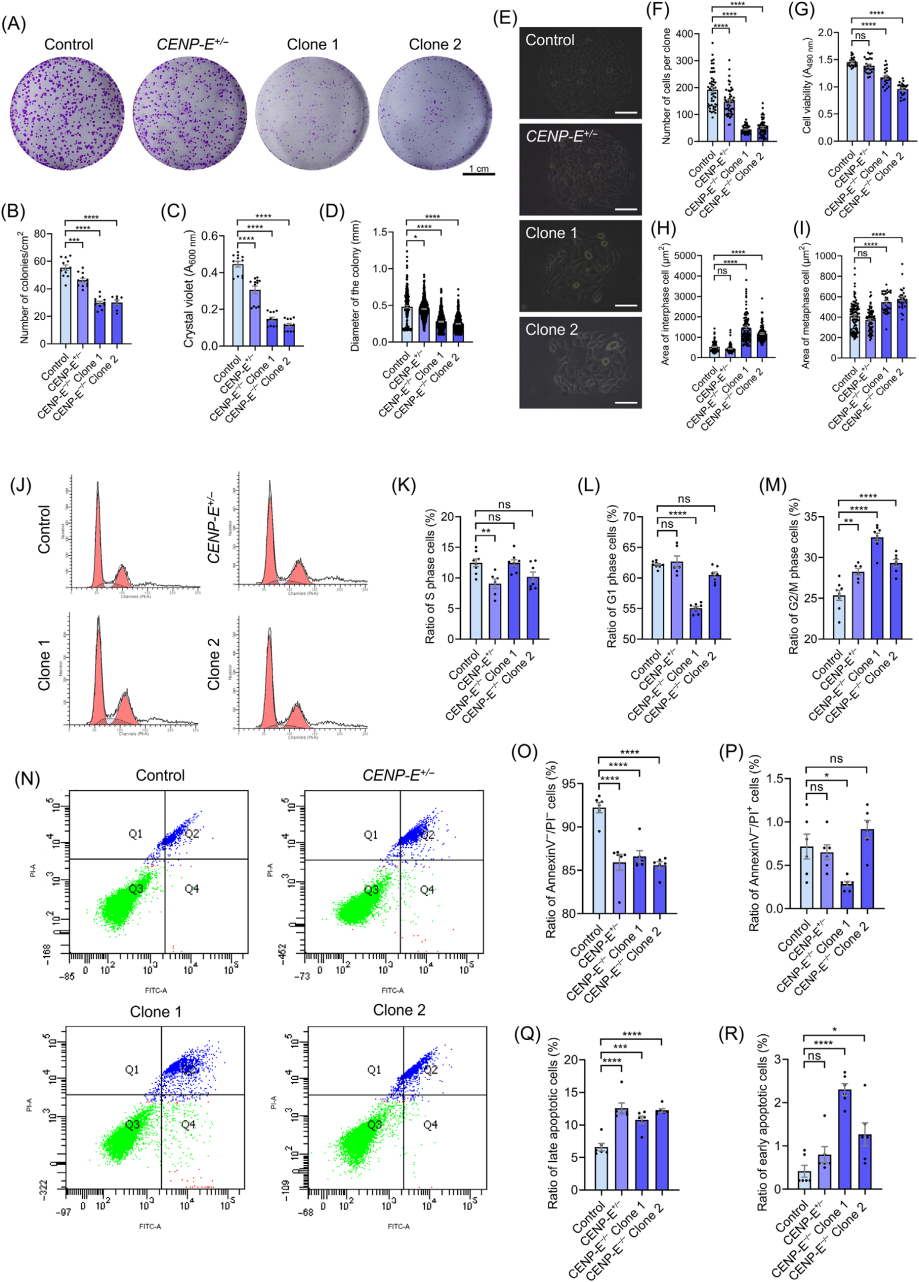
CENP‐E is required for cell proliferation and cell cycle progression in vitro. (A) Crystal violet staining of HeLa cell colony in the control, *CENP‐E* ^*+/−*^, *CENP‐E* ^*−/−*^ Clone 1 and Clone 2 groups. Scale bar, 1 cm. (B) Relative number of cell colonies. Control, *N* = 12. *CENP‐E* ^+/−^, *N* = 12. *CENP‐E* ^−/−^ Clone 1, *N* = 12. *CENP‐E* ^−/−^ Clone 2, *N* = 8. (C) Relative cell viability in the control, *CENP‐E* ^*+/−*^, *CENP‐E* ^*−/−*^ Clone 1 and Clone 2 groups using the crystal violet assay (*A*_600nm_). Control, *N* = 12. *CENP‐E* ^+/−^, *N* = 12. *CENP‐E* ^−/−^ Clone 1, *N* = 12. *CENP‐E* ^−/−^ Clone 2, *N* = 12. (D) The diameters of the cell colony (mm). Control, *N* = 176, group = 3. *CENP‐E* ^+/−^, *N* = 542, group = 3. *CENP‐E* ^−/−^ Clone 1, *N* = 420, group = 3. *CENP‐E* ^−/−^ Clone 2, *N* = 375, group = 3. (E) Representative image of cell colony in the control and CENP‐E knockout groups. Scale bar, 100 μm. (F) The number of cells per clone. Control, *N* = 49. *CENP‐E* ^+/−^, *N* = 51. *CENP‐E* ^−/−^ Clone 1, *N* = 46. *CENP‐E* ^−/−^ Clone 2, *N* = 63. (G) Relative cell viability in the control, *CENP‐E* ^*+/−*^, *CENP‐E* ^*−/−*^ Clone 1 and Clone 2 groups using the MTT assay. Control, *N* = 24, group = 3. *CENP‐E* ^+/−^, *N* = 24, group = 3. *CENP‐E* ^−/−^ Clone 1, *N* = 24, group = 3. *CENP‐E* ^−/−^ Clone 2, *N* = 24, group = 3. (H) The area of interphase cells (μm^2^). Control, *N* = 100. *CENP‐E* ^+/−^, *N* = 102. *CENP‐E* ^−/−^ Clone 1, *N* = 102. *CENP‐E* ^−/−^ Clone 2, *N* = 100. (I) The area of metaphase cells (μm^2^). Control, *N* = 102. *CENP‐E* ^+/−^, *N* = 81. *CENP‐E* ^−/−^ Clone 1, *N* = 34. *CENP‐E* ^−/−^ Clone 2, *N* = 27. (J) Cell cycle analysis of the control, *CENP‐E* ^*+/−*^, *CENP‐E* ^*−/−*^ Clone 1 and Clone 2 groups. (K) The ratios of the S phase cells (%). Control, *N* = 8. *CENP‐E* ^+/−^, *N* = 6. *CENP‐E* ^−/−^ Clone 1, *N* = 8. *CENP‐E* ^−/−^ Clone 2, *N* = 7. (L) The ratios of the G_1_ phase cells (%). Control, *N* = 8. *CENP‐E* ^+/−^, *N* = 6. *CENP‐E* ^−/−^ Clone 1, *N* = 8. *CENP‐E* ^−/−^ Clone 2, *N* = 7. (M) The ratios of the G_2_/M‐phase cells (%). Control, *N* = 8. *CENP‐E* ^+/−^, *N* = 6. *CENP‐E* ^−/−^ Clone 1, *N* = 8. *CENP‐E* ^−/−^ Clone 2, *N* = 7. (N) Cell apoptosis analysis of the control, *CENP‐E* ^*+/−*^, *CENP‐E* ^*−/−*^ Clone 1 and Clone 2 groups. (O) The ratios of Annexin V^−^/PI^−^ cells (%). Control, *N* = 6. *CENP‐E* ^+/−^, *N* = 6. *CENP‐E* ^−/−^ Clone 1, *N* = 6. *CENP‐E* ^−/−^ Clone 2, *N* = 6. (P) The ratios of Annexin V^−^/PI^+^ cells (%). Control, *N* = 6. *CENP‐E* ^+/−^, *N* = 6. *CENP‐E* ^−/−^ Clone 1, *N* = 6. *CENP‐E* ^−/−^ Clone 2, *N* = 6. (Q) The ratios of late apoptotic cells (%). Control, *N* = 6. *CENP‐E* ^+/−^, *N* = 6. *CENP‐E* ^−/−^ Clone 1, *N* = 6. *CENP‐E* ^−/−^ Clone 2, *N* = 6. (R) The ratios of early apoptotic cells (%). Control, *N* = 6. *CENP‐E* ^+/−^, *N* = 6. *CENP‐E* ^−/−^ Clone 1, *N* = 6. *CENP‐E* ^−/−^ Clone 2, *N* = 6. For all graphs, ANOVA Dunnett's multiple comparisons test. ns, *p* > 0.05; *, *p* < 0.05; **, *p* < 0.01; ***, *p* < 0.001; ****, *p* < 0.0001.

We performed the cell colony formation assay and found that *CENP‐E* deletion resulted in a significant decrease in the number of cell colonies in HeLa cells (Figure 7A–C). The diameter of the cell colony also decreased after *CENP‐E* deletion (Figure 7D), indicating that *CENP‐E* deletion led to the suppression of cell proliferation. We also measured cell number in each colony and found that the number of cells per clone decreased in both *CENP‐E* ^*+/−*^ and *CENP‐E* ^*−/−*^ HeLa cells (Figure 7E–G). In addition, the area of interphase and metaphase cells increased in the *CENP‐E* ^*−/−*^ HeLa cells compared with the control (Figure 7H,I). Cell cycle analysis revealed that the ratios of G_2_/M phase cells significantly increased after *CENP‐E* deletion (Figure 7J–M). Meanwhile, *CENP‐E* haploinsufficiency also led to a mild increase in G_2_/M phase cells compared with the control (Figure 7J–M). Cell apoptosis analysis indicated that the ratio of early apoptotic cells and late apoptotic cells increased after *CENP‐E* deletion (Figure 7N–R). Taken together, *CENP‐E* deficiency results in cell cycle arrest, cell growth inhibition and apoptosis in cultured cells.

### 3.4 CENP‐E is required for cell proliferation, cell cycle control and chromosome stability

**FIGURE 8:**
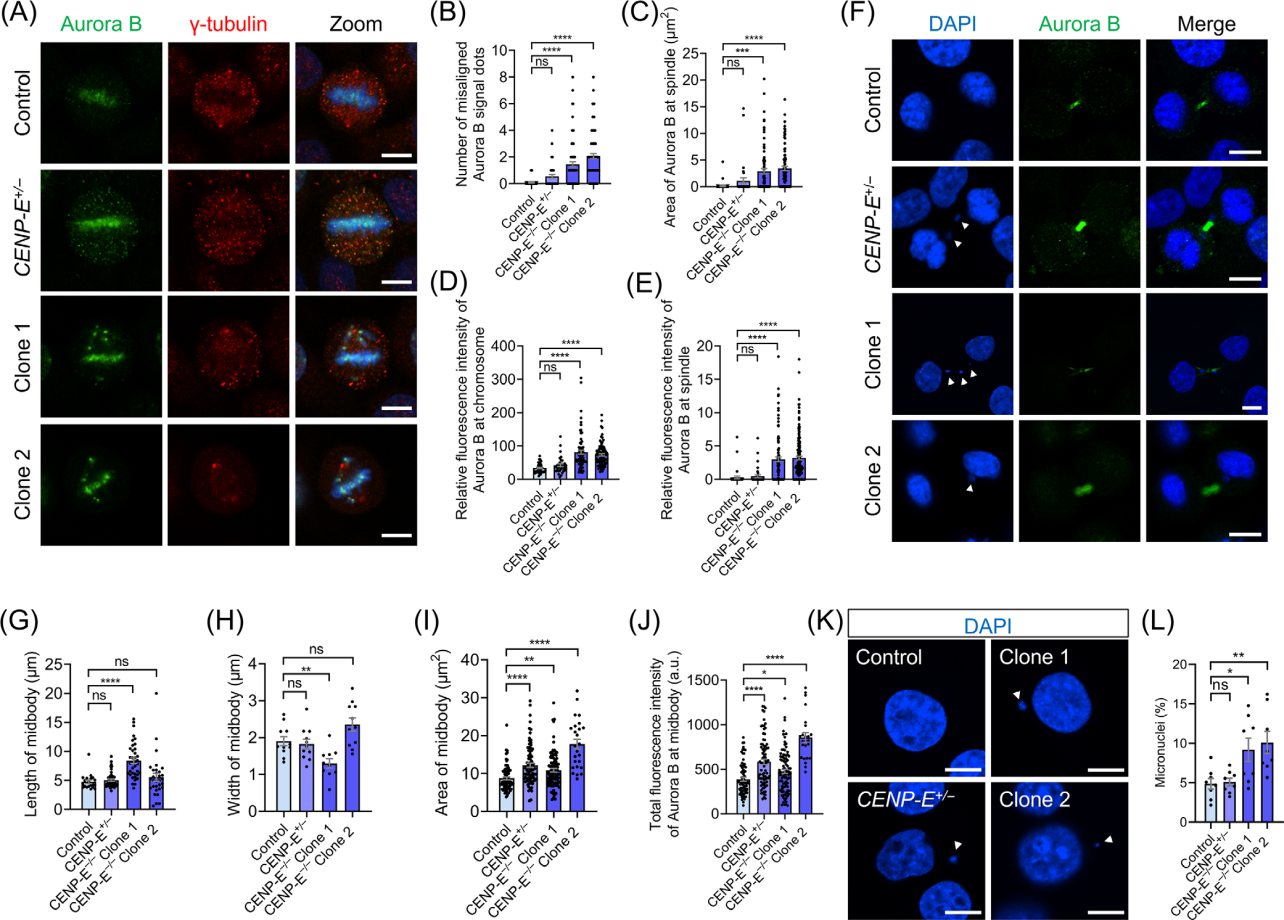
*CENP‐E* deletion resulted in the kinetochore accumulation of Aurora B proteins in metaphase and caused chromosome instability. (A) Immunofluorescence images of γ‐tubulin and Aurora B in the control, *CENP‐E* ^*+/−*^, *CENP‐E* ^*−/−*^ Clone 1 and Clone 2 groups. γ‐tubulin, red; Aurora B, green; DAPI, blue. Scale bar, 10 μm. (B) Quantifications of the number of misaligned Aurora B signal dots. Control, *N* = 43. *CENP‐E* ^+/−^, *N* = 46. *CENP‐E* ^−/−^ Clone 1, *N* = 94. *CENP‐E* ^−/−^ Clone 2, *N* = 91. (C) Area of Aurora B at the spindles (μm^2^). Control, *N* = 37. *CENP‐E* ^+/−^, *N* = 34. *CENP‐E* ^−/−^ Clone 1, *N* = 79. *CENP‐E* ^−/−^ Clone 2, *N* = 86. (D) Relative fluorescence intensity of Aurora B proteins at chromosomes. Control, *N* = 38. *CENP‐E* ^+/−^, *N* = 34. *CENP‐E* ^−/−^ Clone 1, *N* = 79. *CENP‐E* ^−/−^ Clone 2, *N* = 88. (E) Relative fluorescence intensity of Aurora B proteins at the spindles. Control, *N* = 38. *CENP‐E* ^+/−^, *N* = 36. *CENP‐E* ^−/−^ Clone 1, *N* = 102. *CENP‐E* ^−/−^ Clone 2, *N* = 144. (F) Immunofluorescence images of Aurora B proteins at the midbody in the control, *CENP‐E* ^*+/−*^, *CENP‐E* ^*−/−*^ Clone 1 and Clone 2 groups. Aurora B, green; DAPI, blue. Scale bar, 10 μm. (G) Length of the midbody (μm). Control, *N* = 20. *CENP‐E* ^+/−^, *N* = 48. *CENP‐E* ^−/−^ Clone 1, *N* = 38. *CENP‐E* ^−/−^ Clone 2, *N* = 31. (H) Width of the midbody (μm). Control, *N* = 11. *CENP‐E* ^+/−^, *N* = 11. *CENP‐E* ^−/−^ Clone 1, *N* = 10. *CENP‐E* ^−/−^ Clone 2, *N* = 11. (I) Area of the midbody (μm^2^). Control, *N* = 83. *CENP‐E* ^+/−^, *N* = 83. *CENP‐E* ^−/−^ Clone 1, *N* = 94. *CENP‐E* ^−/−^ Clone 2, *N* = 25. (J) Total fluorescence intensity of Aurora B at the midbody (a.u.). Control, *N* = 77. *CENP‐E* ^+/−^, *N* = 81. *CENP‐E* ^−/−^ Clone 1, *N* = 94. *CENP‐E* ^−/−^ Clone 2, *N* = 24. (K) Representative images of micronuclei in the control, *CENP‐E* ^*+/−*^, *CENP‐E* ^*−/−*^ Clone 1 and Clone 2 groups. DAPI, blue. Scale bar, 10 μm. (L) The ratio of micronuclei. Control, *N* = 6959, group = 8. *CENP‐E* ^+/−^, *N* = 7792, group = 8. *CENP‐E* ^−/−^ Clone 1, *N* = 4799, group = 8. *CENP‐E* ^−/−^ Clone 2, *N* = 2857, group = 8. For all graphs, ANOVA Dunnett's multiple comparisons test. ns, *p* > 0.05; *, *p* < 0.05; **, *p* < 0.01; ***, *p* < 0.001; ****, *p* < 0.0001.

To investigate the consequences of chromosome misalignment after *CENP‐E* deletion, we stained HeLa cells with the Aurora B and γ‐tubulin antibodies. We observed that Aurora B proteins remained at the misaligned chromosomes near the spindle poles in *CENP‐E* ^*−/−*^ HeLa cells (Figure 8A,B). Meanwhile, the localization of Aurora B proteins at spindles and chromosomes was influenced after *CENP‐E* deletion (Figure 8A,C–E). Interestingly, we found that in both *CENP‐E* ^*+/−*^ and *CENP‐E* ^*−/−*^ HeLa cells, there were lagging chromosomes and chromosome cuts near the midbody during telophase (Figure 8F). The length, width and area of the midbody were influenced after *CENP‐E* deletion (Figure 8F–I). In addition, the total fluorescence intensity of Aurora B at the midbody increased after *CENP‐E* deletion (Figure 8J). Furthermore, we observed that *CENP‐E* deletion increased micronuclei in both *CENP‐E* ^*+/−*^ and *CENP‐E* ^*−/−*^ HeLa cells (Figure 8K,L).

## 4 DISCUSSION

During cell division, kinesin‐7 CENP‐E is a plus‐end‐directed motor protein required for chromosome biorientation, congression and alignment, which is critical for error‐free chromosome segregation and genome stability.^5^, ^6^, ^10^, ^18^, ^30^, ^44^, ^47^ Genetic deletion of *CENP‐E* results in early embryonic lethal resulting from chromosome misalignment and cell cycle arrest.^26^, ^27^, ^28^, ^48^ In this study, we established three stable *CENP‐E* ^*+/−*^ and *CENP‐E* ^*−/−*^ knockout cell lines using the CRISPR‐Cas9 gene editing and high‐throughput screenings and revealed that *CENP‐E* deletion results in chromosome misalignment and several chromosomes scattered around the spindle poles. During metaphase, BubR1 and Mad1 proteins were enriched on unaligned kinetochores after *CENP‐E* deletion, suggesting that *CENP‐E* completely knockout results in the accumulation of key checkpoint proteins on unattached kinetochores and the activation of the spindle assembly checkpoint. Our results indicate that chromosome misalignment of several chromosomes is sufficient to activate the spindle assembly checkpoint, and induce metaphase arrest and chromosome missegregation in *CENP‐E* ^*−/−*^ HeLa cells. Furthermore, *CENP‐E* deletion leads to lagging chromosomes, the formation of micronuclei and growth retardation in *CENP‐E* ^*−/−*^ HeLa cells, suggesting that the spindle assembly checkpoint is weakened and silenced in the absence of *CENP‐E*.

Our results indicate that CENP‐E inhibition results in chromosome misalignment of many chromosomes, a robust metaphase arrest and cell death, while *CENP‐E* complete knockout results in chromosome misalignment of several chromosomes, a mild metaphase arrest and delayed growth. Consistently, both cells and mice with *CENP‐E* insufficiency develop aneuploidy and chromosome instability in vitro and in vivo, indicating that CENP‐E reduction also leads to missegregation of one or a few chromosomes per cell division, defects in kinetochore‐microtubule attachment and aneuploidy.^20^, ^21^, ^22^, ^23^, ^26^, ^27^, ^28^ Previous studies have shown that high rates of chromosome instability result in cell death through loss of essential chromosomes and tumour suppression.^29^, ^49^, ^50^, ^51^ However, low rates of chromosome instability can promote tumorigenesis through the gain of oncogenes or loss of essential tumour suppressor genes.^29^, ^50^, ^52^, ^53^ These results indicate that CENP‐E is critical for chromosome alignment, the activation and maintenance of spindle assembly checkpoint. CENP‐E inhibition and deletion can lead to errors in chromosome alignment and spindle assembly checkpoint, which contribute to chromosome missegregation. During cell cycle, chromosome missegregation can cause aneuploidy, which is a significant source of genomic instability.

The recruitment of CENP‐E to unattached kinetochores relies on two distinct pathways: the BubR1‐dependent pathway and the pathway dependent on dynein and the outer corona.^17^, ^31^, ^41^, ^43^, ^54^ CENP‐E mediates dynein‐dynactin kinetochore loading for the bidirectional transport of chromosomes.^41^ CENP‐E interacts with BubR1, Mad1, Spindly and the RZZ (ROD, Zwilch and ZW10) complex at the kinetochores through the C‐terminal domain for correct chromosome alignment.^17^, ^54^ In line with previous studies,^31^, ^43^, ^44^, ^45^ our findings demonstrate that *CENP‐E* knockout leads to reduced localization of the BubR1 proteins at the kinetochores during prometaphase, indicating CENP‐E and BubR1 are co‐dependent for their own kinetochore recruitment and checkpoint functions. Interestingly, *CENP‐E* deletion also causes the KIF2C protein to remain on unaligned kinetochores, suggesting an interaction between KIF2C and CENP‐E at the misaligned kinetochores. CENP‐E and the key kinetochore proteins, including BubR1, KIF2C, Aurora B and Mad1, are partly co‐dependent for kinetochore localization and functions. A recent study revealed that Aurora A and B kinases phosphorylate and regulate CENP‐E to promote chromosome congression: Aurora B holds CENP‐E at kinetochore fibres in prometaphase, while Aurora A prevents the accumulation of CENP‐E at the spindle poles.^16^ Further studies are required to reveal the molecular mechanisms for the interactions between CENP‐E and the outer corona proteins in chromosome congression and alignment. In addition, genetic deletion of *CENP‐E* usually results in cell cycle arrest and cell death. The survival of *CENP‐E* knockout cells might be due to several underlying reasons, including potential gene mutations, gene compensations, changes in gene expression profiles or defects in spindle assembly checkpoints, which required to be studied in the future.

In addition, our results reveal that GSK923295‐mediated CENP‐E inhibition resulted in the disassociation of CENP‐E from kinetochores and the accumulation of CENP‐E around spindle poles, indicating a role of CENP‐E in spindle poles. Both CENP‐E inhibition and knockout led to an increase in the proportion of multipolar spindles and centrosome dispersion, suggesting that CENP‐E is associated with bipolar spindle formation and maintenance. The misaligned chromosomes located around the spindle poles after *CENP‐E* inhibition and deletion may influence the organization of the bipolar spindle in metaphase. *CENP‐E* depletion results in abnormal accumulation of pericentriolar material 1 (PCM) and aberrant destabilization of centrosomes, which leads to shortened astral microtubules and oblique cell division.^55^, ^56^ In mammals, CENP‐E plays a conserved role in spindle pole organization and chromosome alignment in diverse species.^9^, ^19^, ^26^, ^43^, ^55^ Most other mammalian species contain mitotic centrioles while the mouse lacks mitotic centrioles during the first cell cycles, so the absence of centrioles might contribute to more readily dispersing centrosomal components. CENP‐E may exert a microtubule crosslinking and sliding force in interpolar spindles and result in a force imbalance at spindle poles,^19^, ^47^, ^57^ which remains to be clarified in future. These results provided evidence that CENP‐E is essential for spindle microtubule organization and the maintenance of spindle poles, which contribute to chromosome congression and alignment in cell division.

## AUTHOR CONTRIBUTIONS

ZYS conceived, designed and supervised the study. JC and ZYS performed the experiments, data analysis, curation, validation and visualization. SW, JJH, YPL, ZYD, HKF, JFC and WYL participate in the investigation, data analysis and interpretation. WYL and ZYS acquired resources and funding. ZYS reviewed and revised the manuscript. All authors read and approved the final manuscript.

## FUNDING INFORMATION

This work was supported by the following grants: National Natural Science Foundation of China (grant numbers 82001608 and 82101678), Natural Science Foundation of Fujian Province, China (grant number 2023J01306), Joint Funds for the Innovation of Science and Technology, Fujian Province (grant number 2023Y9006) and Fujian Medical University high‐level talents scientific research start‐up funding project (grant number XRCZX2017025).

## CONFLICT OF INTEREST STATEMENT

The authors declare no conflicts of interest.

## Supporting information


**Table S1.** Commercialized antibodies used in this study.
**Table S2.** Oligonucleotides and primers used in this study.

**Data S1.** Supporting information.

## Data Availability

The data that support the findings of this study are available from the corresponding author upon reasonable request.
