# Supplementary material for: Kinesin‐7 CENP‐E mediates centrosome organization and spindle assembly to regulate chromosome alignment and genome stability
Source: Cell Prolif. 2024 Sep 12;58(1):e13745. doi: 10.1111/cpr.13745 (PMC11693568; doi:10.1111/cpr.13745)
Supplement: Supplementary file 1 — Table S1. Commercialized antibodies used in this study. Table S2. Oligonucleotides and primers used in this study. [file CPR-58-e13745-s001.docx]

**Supplemental tables, including Table S1 and Table S2.**

**Table S1. Commercialized antibodies used in this study.**

| **Reagent or Resource** | **Source** | **Catalog number** | |
| --- | --- | --- | --- |
| Anti-α-tubulin mouse monoclonal antibody | Abcam | Cat#: ab7291 | |
| Anti-γ-tubulin rabbit monoclonal antibody | Abcam | Cat#: ab179503 |  |
| Anti-γ-tubulin mouse monoclonal antibody | Santa Cruz Biotechnology | Cat#: sc-17788 | |
| Anti-CENP-E rabbit polyclonal antibody | Abcam | Cat#: ab133583 | |
| Anti-CENP-E rabbit polyclonal antibody | Abcam | Cat#: ab124733 | |
| Anti-KIF2C rabbit polyclonal antibody | Sangon Biotech (Shanghai) | Cat#: D225154 | |
| Anti-BubR1 rabbit monoclonal antibody | Abcam | Cat#: ab254326 | |
| Anti-Mad1 rabbit monoclonal antibody | Abcam | Cat#: ab184560 | |
| Anti-pHistone H3 (phoSer 10) rabbit monoclonal antibody | Abcam | Cat#: ab267372 | |
| Anti-Aurora B rabbit monoclonal antibody | Abcam | Cat#: ab45145 | |
| Anti-Aurora A rabbit monoclonal antibody | Abcam | Cat#: ab13824 | |
| Anti-Eg5 rabbit monoclonal antibody | Abcam | Cat#: ab254298 | |
| Alexa Fluor 488-conjugated goat-anti-mouse secondary antibody | Abcam | Cat#: ab150113 | |
| Alexa Fluor 555-conjugated donkey-anti-rabbit secondary antibody | Abcam | Cat#: ab150074 | |
| Alexa Fluor 488-conjugated goat-anti-rabbit secondary antibody | Abcam | Cat#: ab150077 | |
| Alexa Fluor 555-conjugated donkey-anti-mouse secondary antibody | Abcam | Cat#: ab150106 | |

**Table S2. Oligonucleotides and primers used in this study.**

| **Oligonucleotides/Primers** | **Sequence (5’-3’)** | **Applications** |
| --- | --- | --- |
| CRISPR *CENP-E* primer F1 | CTAAGGGAGTCTCCATTGGAGGA | *CENP-E^+/-^* mouse genotyping |
| CRISPR *CENP-E* primer R1 | AGCAACTTGGAACGGAGAAGG | *CENP-E^+/-^* mouse genotyping |
| CRISPR *CENP-E* primer F2 | CCCAAGCATTTGGACTCTGTAC | *CENP-E^+/-^* mouse genotyping |
| CRISPR *CENP-E* primer R2 | TGGGACACTTTCTATGGTGATCT | *CENP-E^+/-^* mouse genotyping |
| CRISPR *CENP-E* primer F3 | GGTCGGGAATGCCAGAAAGA | *CENP-E^+/-^* mouse genotyping |
| CRISPR *CENP-E* primer R3 | CTGCATTGCTAAGTTTGCCACT | *CENP-E^+/-^* mouse genotyping |
| *Stra8 iCre* primer F1 | GGGCAGTCTGGTACTTCCAAGCT | *Stra8 iCre* mouse genotyping |
| *Stra8 iCre* primer R1 | TGAGGCTCTTGCTCCTGACAGAAA | *Stra8 iCre* mouse genotyping |
| *Stra8 iCre* primer F2 | CAGCAAAACCTGGCTGTGGATC | *Stra8 iCre* mouse genotyping |
| *Stra8 iCre* primer R2 | ATGAGCCACCATGTGGGTGTC | *Stra8 iCre* mouse genotyping |
| CRISPR-Cas9 gRNA-1 F1 | CACCGCGGCCGCACTCGCACGCAGA | CRISRP-Cas9 gene editing |
| CRISPR-Cas9 gRNA-1 R1 | AAACTCTGCGTGCGAGTGCGGCCGC | CRISRP-Cas9 gene editing |
| CRISPR-Cas9 gRNA-2 F1 | CACCGTTCTTTAGAGACGCGGGCTC | CRISRP-Cas9 gene editing |
| CRISPR-Cas9 gRNA-2 R1 | AAACGAGCCCGCGTCTCTAAAGAAC | CRISRP-Cas9 gene editing |
| CRISPR-Cas9 target F1 | GAGGGTCCTGGCCATTTTCCTG | *CENP-E* knockout validation |
| CRISPR-Cas9 target R1 | AGATCTCCGATCCTCCCCTGTC | *CENP-E* knockout validation |
| CRISPR-Cas9 target F2 | TGGTAACTGCATTTTGGTGTTCTAC | *CENP-E* knockout validation |
| CRISPR-Cas9 target R2 | CCTGTTGCAACGTGAGGGAAG | *CENP-E* knockout validation |
