# Supplementary material for: Kinesin‐7 CENP‐E mediates centrosome organization and spindle assembly to regulate chromosome alignment and genome stability
Source: Cell Prolif. 2024 Sep 12;58(1):e13745. doi: 10.1111/cpr.13745 (PMC11693568; doi:10.1111/cpr.13745)
Supplement: Supplementary file 2 — Data S1. Supporting information. [file CPR-58-e13745-s002.docx]

**Supplemental Information**

**Kinesin-7 CENP-E mediates centrosome organization and spindle assembly to regulate chromosome alignment and genome stability**

Jie Chen^1,2^, Shan Wu^1,2^, Jie-Jie He^1,2^, Yu-Peng Liu^1,2^, Zhao-Yang Deng^1,2^, Han-Kai Fang^1,2^, Jian-Fan Chen^1,2^, Ya-Lan Wei^3,4^, Zhen-Yu She^1,2,^*

**Affiliations:**

^1^ Department of Cell Biology and Genetics, The School of Basic Medical Sciences, Fujian Medical University, Fuzhou, Fujian, 350122, China.

^2^ Key Laboratory of Stem Cell Engineering and Regenerative Medicine, Fujian Province University, Fuzhou, Fujian, 350122, China.

^3^ Medical Research Center, Fujian Maternity and Child Health Hospital, Fuzhou, Fujian, 350001, China.

^4^ College of Clinical Medicine for Obstetrics & Gynecology and Pediatrics, Fujian Medical University, Fuzhou, Fujian, 350122, China.

***Correspondence should be addressed to** Zhen-Yu She, E-mail: [zhenyushe@fjmu.edu.cn](mailto:zhenyushe@fjmu.edu.cn); Department of Cell Biology and Genetics, The School of Basic Medical Sciences, Fujian Medical University, Fuzhou, Fujian 350122, China. Tel: +86-15750849910.

Supporting information includes Figures S1-S6, Table S1, and Table S2.

**MATERIALS AND METHODS**

**Animals and ethics statements**

The *CENP-E^Flox/+^* mice were purchased from GemPharmatech (Cat# T008554; Jiangsu, China). Through CRISPR/Cas9 technology, the exon 2 region of the *CENP-E* gene (NCBI GenBank accession number NM_175762.4) was designed as a knockout target, and fertilized eggs were obtained through microinjection. F_0_ generation of positive mice was obtained by the transplanted fertilized eggs, which were then mated with the C57BL/6JGpt mice after validation by PCR and Sanger sequencing to obtain a stable F1 mouse model.

The genomic DNA of the mice was extracted using the DNA extraction kit (Cat#B518251; Sangon Biotech). For the *CENP-E* gene, the following primers were used for gene identification, *CENP-E* primer F1: CTAAGGGAGTCTCCATTGGAGGA; *CENP-E* primer R1: AGCAACTTGGAACGGAGAAGG; *CENP-E* primer F2 CCCAAGCATTTGGACTCTGTAC; *CENP-E* primer R2: TGGGACACTTTCTATGGTGATCT; *CENP-E* primer F3: GGTCGGGAATGCCAGAAAGA; *CENP-E* primer R3: CTGCATTGCTAAGTTTGCCACT. The PCR program was listed as follows: 95 ℃ for 3 min; 95 ℃ for 15 s, 60 ℃ for 15 s, 72 ℃ for 60 s, 35 cycles; 72 ℃ for 5 min, and 4 ℃, ∞.

The *Stra8-iCre-ployA* gene was inserted into mouse chromosome 11 by the CRISPR/Cas9 technology. For the *Stra8-iCre* gene, the following primers were used for gene identification, *Stra8-iCre* primer F1: GGGCAGTCTGGTACTTCCAAGCT; *Stra8-iCre* prime R1: TGAGGCTCTTGCTCCTGACAGAAA; *Stra8-iCre* primer F2 CAGCAAAACCTGGGCTGTGGATC; *Stra8-iCre* primer R2: ATGAGCCACCATGTGGGGTGTC. The PCR program was listed as follows: 94 ℃ for 3 min; 94 ℃ for 30 s, 60 ℃ for 30 s, 72 ℃ for 60 s, 35 cycles; 72 ℃ for 5 min, 4 ℃ ∞. Mice were housed in a pathogen-free facility at Fujian Medical University under standard environment exposed to a 12 h: 12 h light: dark cycle with free access to water and food.

**Cell culture, maintenance, and treatment**

Cells were verified to be free of mycoplasma by frequent staining of plated cells with DAPI (Beyotime). Cells were examined for mycoplasma (MycoAlert, Lonza) and authenticated by short tandem repeat (STR) profiling (IdentiCell Molecular Diagnostics). For gene knockout validation, DNA mutations and gene knockout were validated by Sanger sequencing and immunofluorescence. For transfection in each well of a 24-well plate, cells were transfected with 1 μg plasmid using 1.5 μL Lipofectamine 3000 transfection reagent (L3000008; Thermo Fisher Scientific), 50 μL Opti-MEM reduced serum medium (31985062; Thermo Fisher Scientific) according to the manufacturer’s protocols.

For CENP-E RNA interference (RNAi), siRNA knockdown was carried out using RNAi transfection reagent (Sangon, Cat. E607402) following the manufacturer’s guidelines. For *CENP-E* siRNA knockdown, HeLa cells were cultured in the 24-well plate for 24 h, replaced with fresh DMEM medium and transfected at the 60-70% confluent for 24 h. Three independent siRNA oligonucleotides targeting *Homo sapiens* *CENP-E* (GenBank accession number NM_001286734.2; Gene ID: 1062) were synthesized, annealed, purified, and validated according to the manufacturer’s protocols (Sangon Biotech). The sequences of siRNA are listed as follows: negative control, 5’-UUCUCCGAACGUGUCACGUTT-3’; human *CENP-E* siRNA-1, 5’-UUGUCUUGUAUCUCUUUCCTT’, human *CENP-E* siRNA-2, 5’-UUGACUUUCUUGAAGUCUGTT-3’, and human *CENP-E* siRNA-3, 5’-UUUAUCAACAGUUUCUUUGTT-3’. For transfection control, FAM siRNA, 5’- (FAM)UUCUCCGAACGUGUCACGUTT-3’; For positive control, GAPDH siRNA, 5’-GUAUGACAACAGCCUCAAGTT-3’.

For a 24-well plate, 3 μl RNA TransMate reagent (Sangon, Cat. E607402) was diluted into a 195 μl serum-free Opti-MEM medium (Thermo Fisher Scientific, Cat. 31985070). A 2 μl 20 μM siRNA was then diluted into a 200 μl serum-free Opti-MEM medium. The siRNA and TransMate reagents were gently mixed at room temperature for 10 min, and then added into cells at a final concentration at 40 nM. Cells were transfected with negative control, *CENP-E* siRNA-1, *CENP-E* siRNA-2 and *CENP-E* siRNA-3 at 37℃ for 24 h, respectively. Cells were harvested at 24 h after siRNA transfection for further analysis. The efficiency of *CENP-E* siRNA knockdown was validated according to immunohistochemistry and the manufacturer’s protocols.

**Hematoxylin-Eosin (HE) staining**

For HE staining, tissues were harvested and fixed in 10% formaldehyde at room temperature for 16 h. The tissues were dehydrated in 50% ethanol for 30 min, in 70% ethanol for 30 min, in 85% ethanol for 30 min, in 95% ethanol for 30 min, and in 100% ethanol for 1 h. Tissues were incubated with xylene for 40 min and then in paraffin at 65 ℃ for 2 h. The 5-μm-thick slides were cut using an ultra-thin semiautomatic microtome (Cat# RM2016; Leica). The slides were immersed with xylene for 40 min, dehydrated in 100% ethanol for 6 min, 95% ethanol for 2 min, 80% ethanol for 2 min, and then 70% ethanol for 2 min. Slides were incubated with distilled water for 2 min and then stained with Mayer’s hematoxylin solution at room temperature for 4 min. Slides were incubated with running water for 6 min and then with distilled water for 2 min. Slides were incubated with 1% ethanol hydrochloride for 3 s and then washed with running water for 1 min. Slides were stained with 1% eosin solution for 50 s and washed two times with 95% ethanol for 10 s. Slides were incubated with 100% ethanol for 3 min and then with xylene for 40 min. Slides were sealed with neutral gum for further analysis.

**Immunofluorescence and confocal microscopy**

For tissue paraffin slides, the 5-μm-thick slides were deparaffinized in xylene for 40 min and rehydrated in gradient ethanol, anhydrous ethanol for 6 min, 95% ethanol for 2 min, 80% ethanol for 2 min, and 70% ethanol for 2 min. Slides were incubated with distilled water for 2 min and then in PBS for 2 min. For antigen retrieval, slides were incubated with 10 mM citrate buffer (pH 6.0) and boiled for 3 min according to the standard high-pressure repair method. In the negative control groups for antigen retrieval, the primary antibody was omitted after antigen retrieval to confirm that the fluorescence was antibody-specific. For antigen retrieval specificity, the control without heat treatment was applied to validate non-specific epitopes. For primary antibody control, no primary antibody was used to validate the specificity of the secondary antibodies. Slides were blocked with 3% BSA in PBST for 1 h and then incubated with primary antibodies at 4 ℃ for 16 h. Samples were rinsed three times with PBS and then incubated with secondary antibodies at 37 ℃ for 1 h. Samples were rinsed with PBS three times, stained with DAPI for 5 min, and then mounted with an anti-fade mounting medium.

**Western Blot**

For western blot analysis, total proteins of the HeLa cells were extracted using the SDS lysis buffer (Beyotime, Cat. P0013G) at 4 ℃ for 20 min according to manufacturer’s protocols. Total proteins were separated by 10% polyacrylamide gels (Beyotime, Cat. P0917S) at 100 V for 3-4 h and were transferred onto polyvinylidene difluoride (PVDF) membranes (GE, Cat. 10600023) at 200 mA for 90 min. After being incubated in 5% non-fat milk in TBST buffer at room temperature for 1 h, PVDF membranes were rinsed with TBST for 5 min. Samples were incubated with the primary antibody at 4 ℃ for 12 h and then rinsed with TBST for 5 min three times. Samples were incubated with HRP-conjugated goat anti-rabbit IgG (Immunoway, Cat. RS0002, 1:3000) or HRP-conjugated goat anti-mouse IgG (Immunoway, Cat. RS0001). Protein bands were visualized and analyzed using the BeyoECL Star kit (Beyotime, Cat. P0018AFT). The western blot images were recorded using a chemiluminescent imaging analysis system (Tanon, No. 5200).

**The TUNEL assay**

For TUNEL analysis, 5-μm-thick slides were incubated with xylene for 20 min, with anhydrous ethanol for 5 min, 90% ethanol for 2 min, 70% ethanol for 2 min, and in distilled water for 2 min. Samples were incubated with 20 μg/mL DNase-free proteinase K (Cat#, Sangon Biotech) in 10mM Tris-HCl (pH 7.4) at 37 ℃ for 30 min. Slides were rinsed with PBS three times and then stained with fluorescein isothiocyanate (FITC)-dUTP and the TdT-mediated dUTP Nick-End Labeling (TUNEL) solution (Cat# C1086; Beyotime) according to the manufacturer’s protocols. Slides were rinsed with PBS three times and then stained with DAPI for 5 min. Slides were mounted with an anti-fade mounting medium and recorded using a Leica scanning confocal microscope (Leica No. TCS SP8) equipped with an HC PL APO CS2 63×/NA 1.40 objective.

**Transmission Electron Microscopy**

Cells were digested with 0.25% Trypsin-EDTA at 37 ℃ for 1 min and then centrifuged at 1000 × g at 37 ℃ for 5 min. Cells were fixed in 3% glutaraldehyde-1.5% paraformaldehyde in 0.1 M PBS (pH 7.2) at room temperature for 24 h. Cells were fixed in 1% osmic acid-1.5% potassium ferrocyanide at 4 °C for 1.5 h. Cells were washed three times with 0.1 M PBS for 5 min. Cells were incubated with 50% ethanol for 10 min, and then in 70% ethanol-saturated uranium acetate dye at 4 °C for 12 h. Cells were incubated with 90% ethanol for 10 min, 90% ethanol-acetone for 10 min, and then anhydrous acetone for 10 min three times. Cells were incubated with anhydrous acetone-epoxy resin 618 embedding agent for 90 min and then with epoxy resin 618 embedding agent at 35 °C for 3 h. Samples were incubated with epoxy resin 618 embedding agent at 35 °C for 12 h, at 45 °C for 12 h, and at 60 °C for 24 h. The 90 nm-thick slides were cut using an ultra-thin microtome (Cat# EM UC-7; Leica), stained with uranyl acetate for 10 min, and then stained with lead citrate for 10 min. Images were acquired using a transmission electron microscope (Cat# Tecnai G2; FEI) and affinity software.

**Statistical analysis**

Images were processed using ImageJ and affinity designer software. All cells were selected from nonoverlapping regions. For quantifications of the fluorescence intensity, signal intensity was quantified using ImageJ. The signal of the same area at the cytoplasm was subtracted as background signals. Sample size, statistical tests, and number of replicates are indicated in each figure legend. No statistical methods were used to predetermine the sample size. The experiments were not randomized and the investigators were not blinded to allocation during experiments and outcome assessment. For cell apoptosis analysis, Annexin V-FITC^-^/PI^-^ cells indicate normal cells. Annexin V-FITC^+^/PI^-^ cells indicate early apoptotic cells. Annexin V-FITC^+^/PI^+^ cells indicate late apoptotic cells or necrotic cells.

**FIGURE S1**


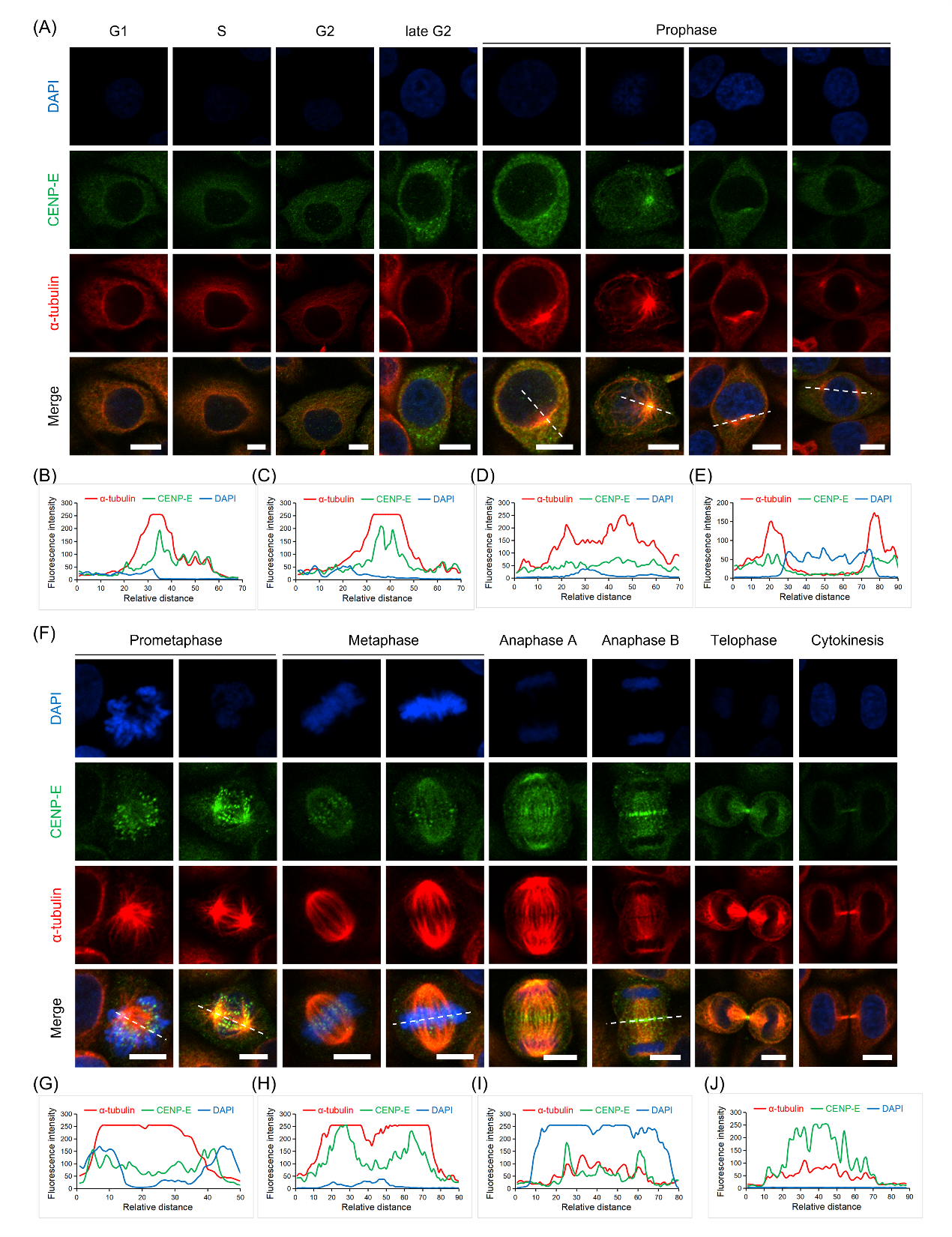


**FIGURE S1** Dynamic localization of CENP-E proteins in the cell cycle. (a) Immunofluorescence images of α-tubulin and CENP-E in HeLa cells from G_1_ phase to prophase. α-tubulin, red; CENP-E, green; DAPI, blue. Scale bar, 10 μm. (b-e) Line-scan analyses of fluorescence intensity of α-tubulin and CENP-E in HeLa cells during prophase. The X-axis indicates the relative distance. The Y-axis indicates the fluorescence intensity. (f) Immunofluorescence images of α-tubulin and CENP-E in HeLa cells from prometaphase to cytokinesis. α-tubulin, red; CENP-E. green; DAPI, blue. Scale bar, 10 μm. (g-j) Line-scan analyses of fluorescence intensity of α-tubulin and CENP-E in HeLa cells. The X-axis indicates the relative distance. The Y-axis indicates the fluorescence intensity.

**FIGURE S2**


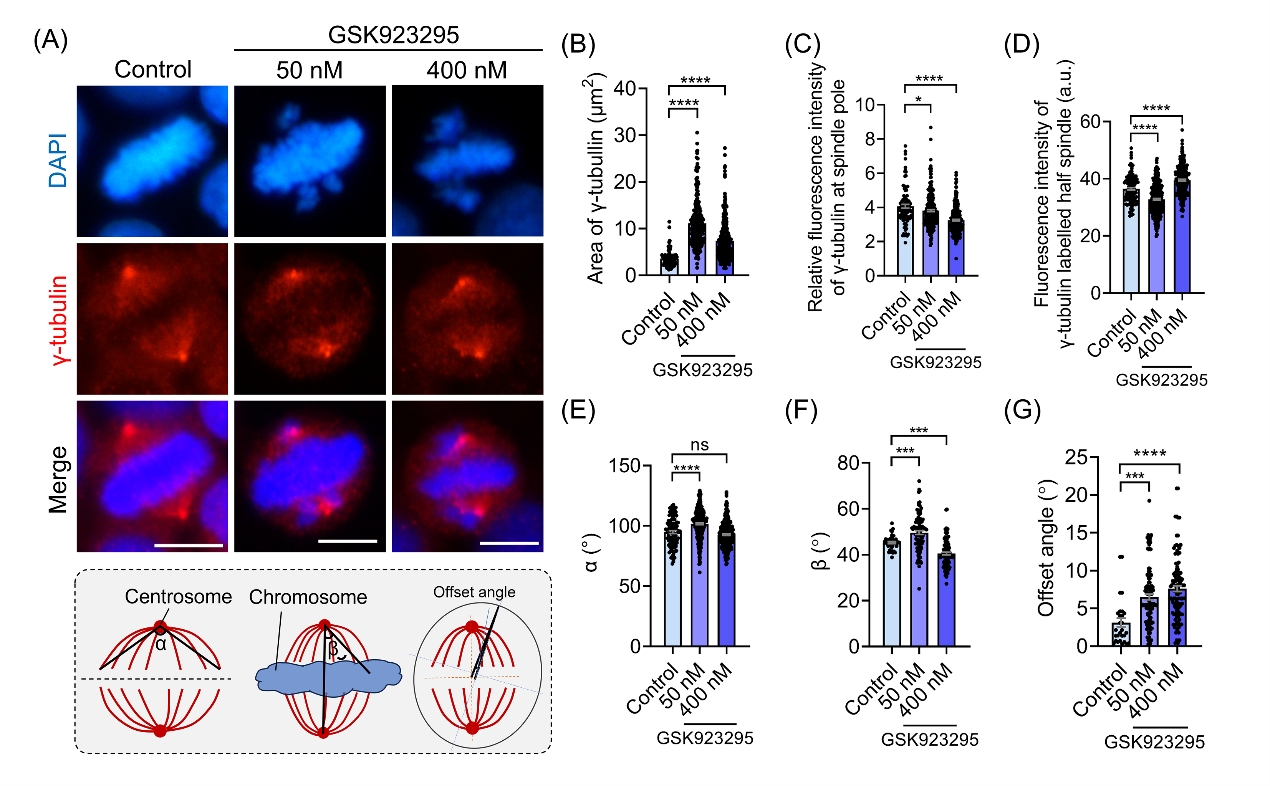


**FIGURE S2** CENP-E regulates the organization of spindle poles and the maintenance of the centrosome. (A) Immunofluorescence images of γ-tubulin in the control and GSK923295 groups. γ-tubulin, red; DAPI, blue. Scale bar, 10 μm. The measurement index of α-angle and β-angle values were shown. (B) The area of γ-tubulin proteins (μm^2^). Control, N = 101. GSK923295 50 nM, N = 282. GSK923295 400 nM, N = 316. (C) Relative fluorescence intensity of γ-tubulin at the spindle poles. Control, N = 99. GSK923295 50 nM, N = 282. GSK923295 400 nM, N = 316. (D) Fluorescence intensity of γ-tubulin-labeled half spindle (arbitrary unit, a.u.). Control, N = 122. GSK923295 50 nM, N = 312. GSK923295 400 nM, N = 311. (E) Quantifications of the α-angle. Control, N = 105. GSK923295 50 nM, N = 334. GSK923295 400 nM, N = 369. (F) Quantifications of the β-angle. Control, N = 43. GSK923295 50 nM, N = 93. GSK923295 400 nM, N = 95. (G) The offset angle values. The offset angle indicates the angle between the cell center and the center of the equatorial plate. Control, N = 28. GSK923295 50 nM, N = 114. GSK923295 400 nM, N = 120. For all graphs, mean ± SEM. For all graphs, ANOVA Dunnett’s multiple comparisons test. ns, *P* > 0.05; *, *P* < 0.05; **, *P* < 0.01; ***, *P* < 0.001; ****, *P* < 0.0001.

**FIGURE S3**


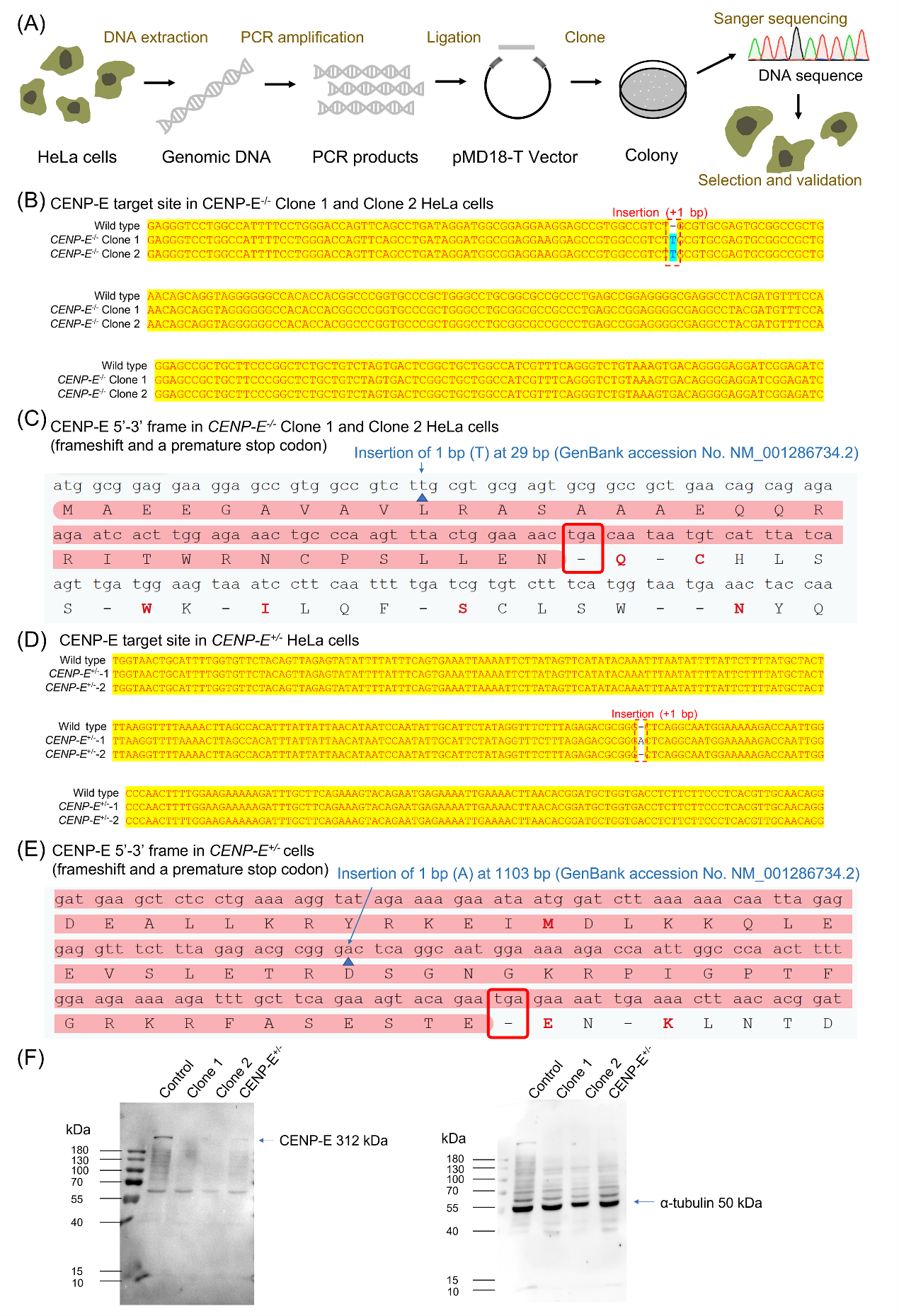


**FIGURE S3** Construction and validation of *CENP-E^+/-^* and *CENP-E^-/-^* HeLa cells using the CRISPR-Cas9 gene editing system and Sanger sequencing. (A) Schematic for the construction and selection of *CENP-E* knockout HeLa cells using the CRISPR-Cas9 system. (B) Multiple sequence alignment of the CENP-E target sites in wild-type, *CENP-E^-/-^* Clone 1 and Clone 2 cells. The DNA sequence of PCR products amplified from the *CENP-E* gene of the *CENP-E^-/-^* HeLa cells was shown. There is a 1 bp insertion of T at the position 29 bp in the coding region of *CENP-E* cDNA (NM_001286734.2). These results in complete gene knockout of *CENP-E* in the *CENP-E^-/-^* cells. (C) Frameshift and a premature translation stop in the *CENP-E^-/-^* Clone 1 and Clone 2 cells. (D) Multiple sequence alignment of the CENP-E target sites in wild type and *CENP-E^+/-^* cells. There is a 1 bp insertion of A at the position 1103 bp in the coding region of CENP-E cDNA (NM_001286734.2). These results in heterozygous mutation of CENP-E in *CENP-E^+/-^* cells. (E) Frameshift and a premature translation stop in one CENP-E DNA locus of the *CENP-E^+/-^* cells. (F) Western Blot analysis of CENP-E and α-tubulin in the wild-type, *CENP-E^-/-^* Clone 1, *CENP-E^-/-^* Clone 2 cells and *CENP-E^+/-^* HeLa cells. CENP-E, 312 kDa. α-tubulin, 50 kDa. Western blot showing that the expression level of CENP-E proteins was completely knockout in the *CENP-E^-/-^* Clone 1 (Lane 2) and *CENP-E^-/-^* Clone 2 cells (Lane 3) compared with the wild-type HeLa cells (Control, Lane 1). In addition, the expression level of CENP-E protein was significantly decreased in the *CENP-E^+/-^* cells (Lane 4). The same PVDF membrane was washed and re-stained with the anti-α-tubulin antibody to confirm the equal protein loading. α-tubulin serves as the loading control. Molecular weight markers are indicated on the left side of the blot.

**FIGURE S4**


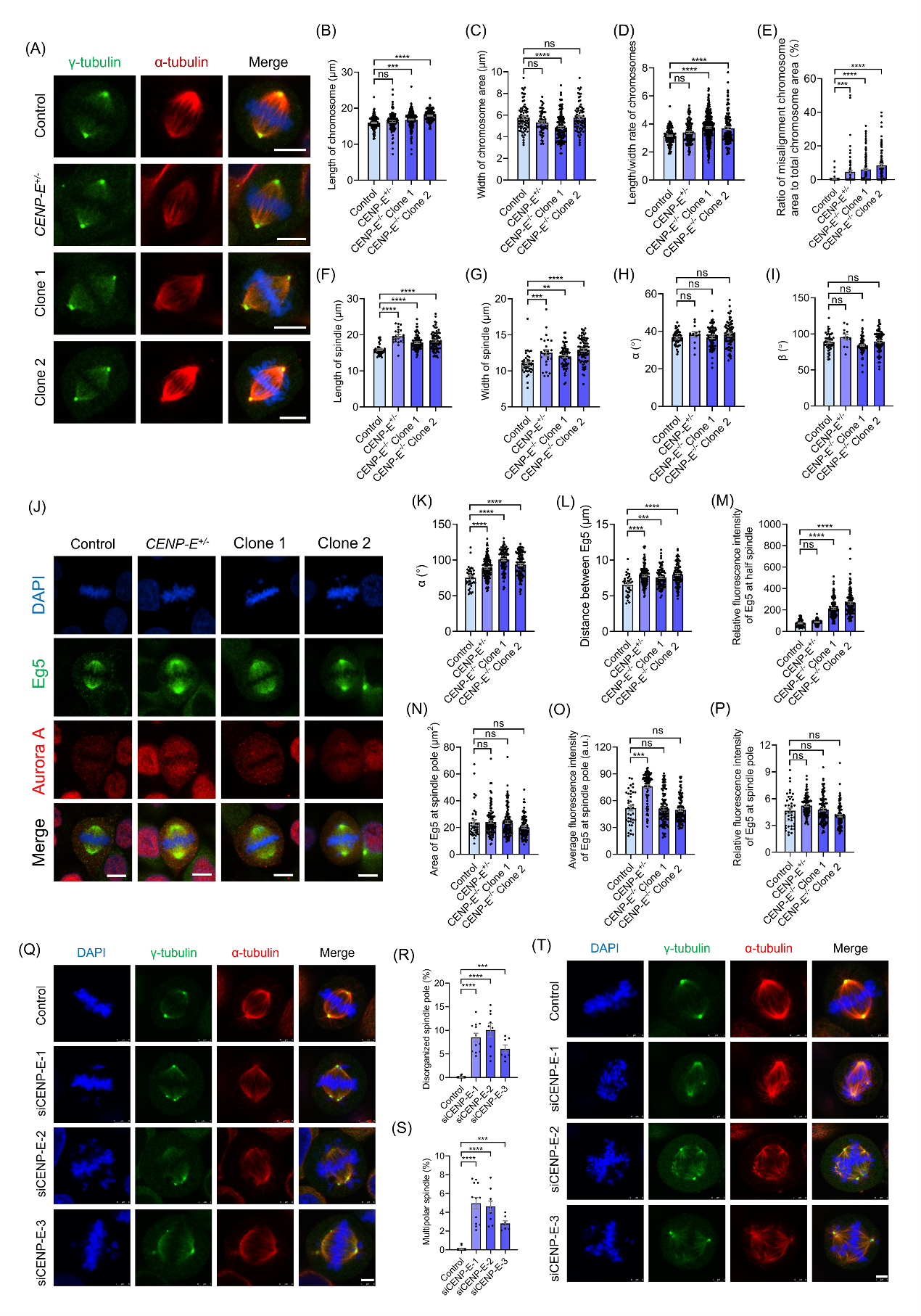


**FIGURE S4** CENP-E is essential for spindle organization and the localization of Eg5 and Aurora A proteins. (A) Immunofluorescence images of α-tubulin and γ-tubulin in the control, *CENP-E^+/-^*, *CENP-E^-/-^* Clone 1 and Clone 2 groups. α-tubulin, red; γ-tubulin, green; DAPI, blue. Scale bar, 10 μm. (B) Length of aligned chromosomes. Control, N = 264. *CENP-E*^+/-^, N = 245. *CENP-E*^-/-^ Clone 1, N = 569. *CENP-E*^-/-^ Clone 2, N = 295. (C) Width of aligned chromosomes. Control, N = 264. *CENP-E*^+/-^, N = 245. *CENP-E*^-/-^ Clone 1, N = 569. *CENP-E*^-/-^ Clone 2, N = 295. (D) Quantifications of the length/width rate of aligned chromosomes. Control, N = 264. *CENP-E*^+/-^, N = 245. *CENP-E*^-/-^ Clone 1, N = 569. *CENP-E*^-/-^ Clone 2, N = 295. (E) The ratios of the misaligned chromosome area to the total chromosome area. Control, N = 111. *CENP-E*^+/-^, N = 83. *CENP-E*^-/-^ Clone 1, N = 140. *CENP-E*^-/-^ Clone 2, N = 92. (F) Length of mitotic spindle. Control, N = 40. *CENP-E*^+/-^, N = 24. *CENP-E*^-/-^ Clone 1, N = 80. *CENP-E*^-/-^ Clone 2, N = 78. (G) Width of mitotic spindle. Control, N = 40. *CENP-E*^+/-^, N = 24. *CENP-E*^-/-^ Clone 1, N = 74. *CENP-E*^-/-^ Clone 2, N = 82. (H) Quantifications of the α-angle values. Control, N = 46. *CENP-E*^+/-^, N = 12. *CENP-E*^-/-^ Clone 1, N = 76. *CENP-E*^-/-^ Clone 2, N = 74. (I) The β-angle values. Control, N = 46. *CENP-E*^+/-^, N = 12. *CENP-E*^-/-^ Clone 1, N = 76. *CENP-E*^-/-^ Clone 2, N = 72. (J) Quantifications of the α-angle values of Eg5 proteins in the control and *CENP-E* knockout groups. Control, N = 39. *CENP-E*^+/-^, N = 128. *CENP-E*^-/-^ Clone 1, N = 100. *CENP-E*^-/-^ Clone 2, N = 115. (K) Quantifications of the α-angle values of Eg5 proteins in the control and *CENP-E* knockout groups. Control, N = 39. *CENP-E^+/-^*, N = 128. *CENP-E^-/-^* Clone 1, N = 100. *CENP-E^-/-^* Clone 2, N = 115. (L) Distances between Eg5 proteins at two spindle poles (μm). Control, N = 34. *CENP-E*^+/-^, N = 128. *CENP-E*^-/-^ Clone 1, N = 94. *CENP-E*^-/-^ Clone 2, N = 109. (M) Relative total fluorescence intensity of Eg5 proteins at the half spindle (FL _half spindle_/FL _cytoplasm_). Control, N = 48. *CENP-E*^+/-^, N = 77. *CENP-E*^-/-^ Clone 1, N = 123. *CENP-E*^-/-^ Clone 2, N = 120. (N) The area of the fluorescence of Eg5 proteins at spindle poles (μm^2^). Control, N = 39. *CENP-E*^+/-^, N = 98. *CENP-E*^-/-^ Clone 1, N = 113. *CENP-E*^-/-^ Clone 2, N = 96. (O) Average fluorescence intensity of Eg5 proteins at the spindle pole (a.u.). Control, N = 39. *CENP-E*^+/-^, N = 98. *CENP-E*^-/-^ Clone 1, N = 113. *CENP-E*^-/-^ Clone 2, N = 96. (P) Relative average fluorescence intensity of Eg5 proteins at the spindle pole. Control, N = 39. *CENP-E*^+/-^, N = 98. *CENP-E*^-/-^ Clone 1, N = 113. *CENP-E*^-/-^ Clone 2, N = 96. (Q) Immunofluorescence images of α-tubulin and γ-tubulin in the control, siCENP-E-1, siCENP-E-2 and siCENP-E-3 groups. α-tubulin, red; γ-tubulin, green; DAPI, blue. Scale bar, 5 μm. *CENP-E* knockdown results in the disorganized spindle poles and dispersed γ-tubulin signals at the centrosomes. Three *CENP-E* specific siRNAs were transfected into the wild-type HeLa cells for 24 h and then harvested for further analysis. Negative control serves as the control. (R) The ratios of disorganized spindle poles in the control, siCENP-E-1, siCENP-E-2 and siCENP-E-3 groups. Control, 0.09 ± 0.06%, N = 2110. *CENP-E*^+/-^, 8.50 ± 0.89%, N = 1173. *CENP-E*^-/-^ Clone 1, 10.07 ± 1.43%, N = 1032. *CENP-E*^-/-^ Clone 2, 6.08 ± 0.84%, N = 989. (S) The ratios of multipolar spindle in the control, siCENP-E-1, siCENP-E-2 and siCENP-E-3 groups. Control, 0.09 ± 0.06%, N = 2110. *CENP-E*^+/-^, 4.96 ± 0.62%, N = 1173. *CENP-E*^-/-^ Clone 1, 4.62 ± 0.59%, N = 1032. *CENP-E*^-/-^ Clone 2, 2.81 ± 0.27%, N = 989. (T) Immunofluorescence images of α-tubulin and γ-tubulin in the control, siCENP-E-1, siCENP-E-2 and siCENP-E-3 groups. The phenotype of multipolar spindle was shown. *CENP-E* knockdown results in the formation of multipolar spindle in siCENP-E-1, siCENP-E-2 and siCENP-E-3 groups compared with control. α-tubulin, red; γ-tubulin, green; DAPI, blue. Scale bar, 5 μm. For all graphs, ANOVA Dunnett’s multiple comparisons test. ns, *P* > 0.05; *, *P* < 0.05; **, *P* < 0.01; ***, *P* < 0.001; ****, *P* < 0.0001.

**FIGURE S5**


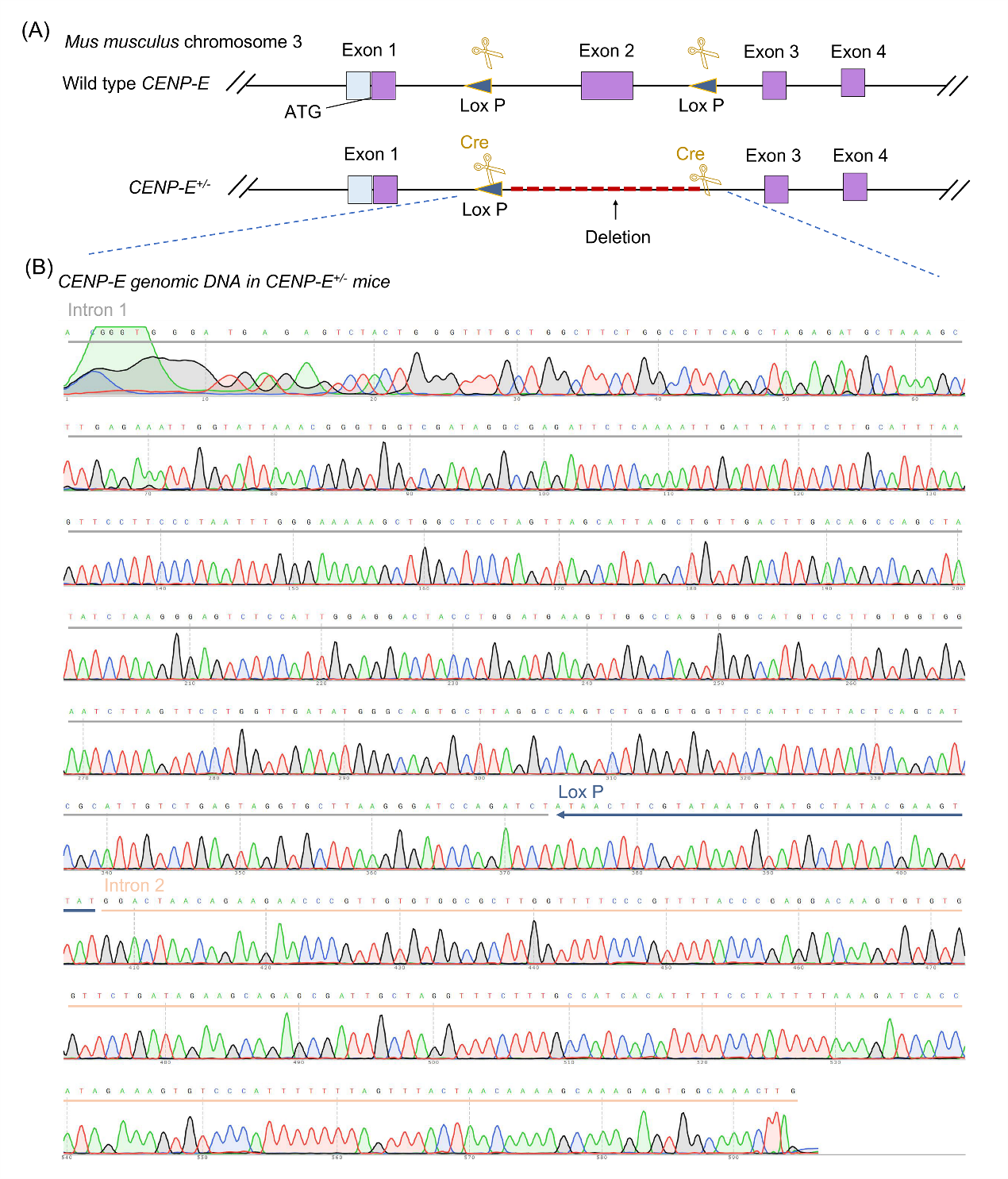


**FIGURE S5** Construction and validation of the *CENP-E^+/-^* mice using the CRISPR-Cas9 and Cre-LoxP system. (A) Schematic of the *CENP-E* gene editing in the *CENP-E^+/-^* C57BL mice using the Cre-LoxP system. Two LoxP sites were induced into intron 1 and intron 3 of CENP-E DNA. The Cre induces a genomic deletion of Exon2 in mouse CENP-E DNA, which results in a frameshift and a premature stop codon. (B) DNA sequence of PCR products amplified from the CENP-E gene of a heterozygous mutant *CENP-E^+/-^* mouse.

**FIGURE S6**


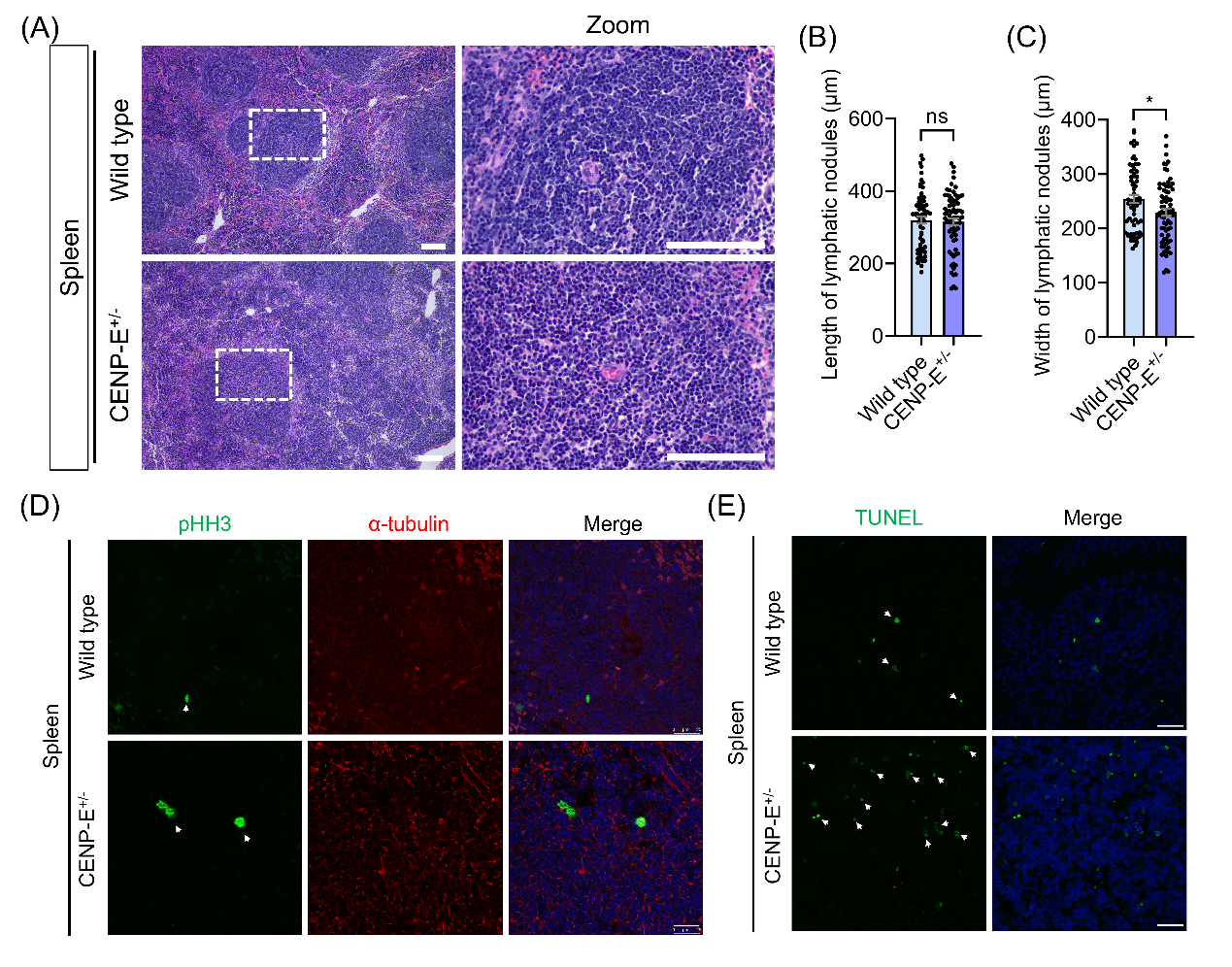


**FIGURE S6** *CENP-E* heterozygous mutation slightly influences the development of the spleen and eye. (A) HE staining of the splenic corpuscle in the spleen of the wild-type and *CENP-E^+/-^* mice. Scale bar, 100 μm. The splenic corpuscle was shown in the zoom. Scale bar, 100 μm. (B) Length of lymphatic nodules (μm). Wild type, N = 66. *CENP-E*^+/-^, N = 74. (C) Width of lymphatic nodules (μm). Wild type, N = 66. *CENP-E*^+/-^, N = 74. (D) Representative immunofluorescence images of pHH3 and α-tubulin in the splenic corpuscle of the spleen in the wild-type and *CENP-E^+/-^* mice. Scale bar, 25 μm. (E) Representative TUNEL staining of the eye in the wild-type and *CENP-E^+/-^* mice. Scale bar, 25 μm. For all graphs, unpaired Student’s *t*-test. ns, *P* > 0.05; *, *P* < 0.05.
